# The interplay of mitophagy, autophagy, and apoptosis in cisplatin-induced kidney injury: involvement of ERK signaling pathway

**DOI:** 10.1038/s41420-024-01872-0

**Published:** 2024-02-24

**Authors:** Iva Suman, Lidija Šimić, Gordana Čanadi Jurešić, Sunčica Buljević, Damir Klepac, Robert Domitrović

**Affiliations:** 1 Department of Medical Chemistry, Biochemistry and Clinical Chemistry, Faculty of Medicine, University of Rijeka, Rijeka, Croatia; 2 Point-of-Care Laboratory, Emergency Department Sušak, Clinical Hospital Center Rijeka, Rijeka, Croatia; 3 Centre for Micro- and Nanosciences and Technologies, University of Rijeka, Rijeka, Croatia

**Keywords:** Mitophagy, Apoptosis

## Abstract

AKI induced by CP chemotherapy remains an obstacle during patient treatments. Extracellular signal-regulated protein kinases 1/2 (ERK), key participants in CP-induced nephrotoxicity, are suggested to be involved in the regulation of mitophagy, autophagy, and apoptosis. Human renal proximal tubular cells (HK-2) and BALB/cN mice were used to determine the role of ERK in CP-induced AKI. We found that active ERK is involved in cell viability reduction during apoptotic events but exerts a protective role in the early stages of treatment. Activation of ERK acts as a maintainer of the mitochondrial population and is implicated in mitophagy initiation but has no significant role in its conduction. In the late stages of CP treatment when ATP is deprived, general autophagy that requires ERK activation is initiated as a response, in addition to apoptosis activation. Furthermore, activation of ERK is responsible for the decrease in reserve respiratory capacity and controls glycolysis regulation during CP treatment. Additionally, we found that ERK activation is also required for the induction of NOXA gene and protein expression as well as FoxO3a nuclear translocation, but not for the regular ERK-induced phosphorylation of FoxO3a on Ser294. In summary, this study gives detailed insight into the involvement of ERK activation and its impact on key cellular processes at different time points during CP-induced kidney injury. Inhibitors of ERK activation, including Mirdametinib, are important in the development of new therapeutic strategies for the treatment of AKI in patients receiving CP chemotherapy.

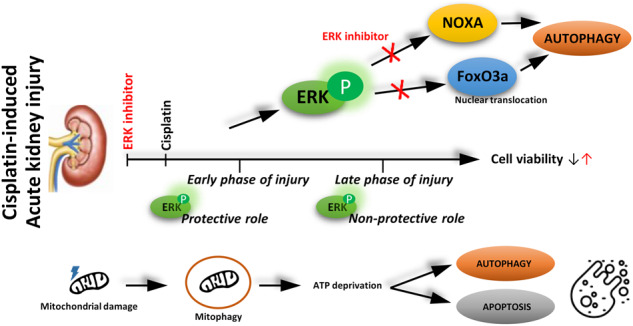

## Introduction

The platinum drug cisplatin (CP) is routinely used in the treatment of solid tumors but its application results in numerous side effects [1]. Clinically, CP’s toxicity to the kidneys is the main obstacle to its use [2]. Acute kidney injury (AKI) remains a common complication of CP chemotherapy, despite extensive use of hydration in clinical practice [3]. The exact mechanism of CP-mediated nephrotoxicity is not yet elucidated despite numerous efforts. Mitochondrial damage, indicated structurally and functionally by decreased cellular respiration and ATP production, is recognized as one of the main events causing renal tubular injury [4]. The importance of mitochondria in cell metabolism and survival makes this organelle an appealing target for extracellular regulated kinases 1/2 (ERK)-dependent cell destiny studies [5]. In our previous studies, we have shown that p-ERK is upregulated in CP-induced AKI in vivo [6, 7] and in vitro [8]. Activated ERK is found in mitochondrial fractions after CP treatment, and it may contribute to enhanced mitochondrial membrane potential, decreased oxidative phosphorylation, and increased apoptosis [9]. Studies using pharmacological inhibitors of mitogen-activated protein kinase 1/2 (MEK) have shown that ERK affects mitochondrial activities, especially those linked with cell death. However, the involvement of ERK mechanistically in the regulation of mitophagy, autophagy, and apoptosis in CP-induced kidney injury has not been clarified. Autophagy is well-known to be activated during CP nephrotoxicity [10] and the autophagic process and lysosomal gene expression can be regulated by ERK signaling [5]. Furthermore, ERK activation can be implicated in mitophagy [11], a selective type of autophagy in which malfunctioning mitochondria are degraded to preserve a healthy mitochondrial population [12], a process activated during CP nephrotoxicity [13]. Furthermore, ERK has been shown to mediate mitochondria-dependent apoptotic [14] signaling and act as an upstream signal for caspase 3-mediated apoptosis during CP-induced acute renal failure in mice [15]. For the first time, we determined the timeline of events during CP nephrotoxicity and defined mechanisms. ERK activation and localization during CP injury are strongly involved in the determination of cell fate and the shift from mitophagy to autophagy, and apoptosis in vitro and in vivo. Overall, our results show that in CP-nephrotoxicity, ERK is implicated in its progression by targeting multiple critical mechanisms in kidney damage and is a potential target to overcome nephrotoxicity in patients receiving CP chemotherapy.

## Results

### ERK is involved in the viability decrease of renal tubular epithelial cells

**Fig. 1:**
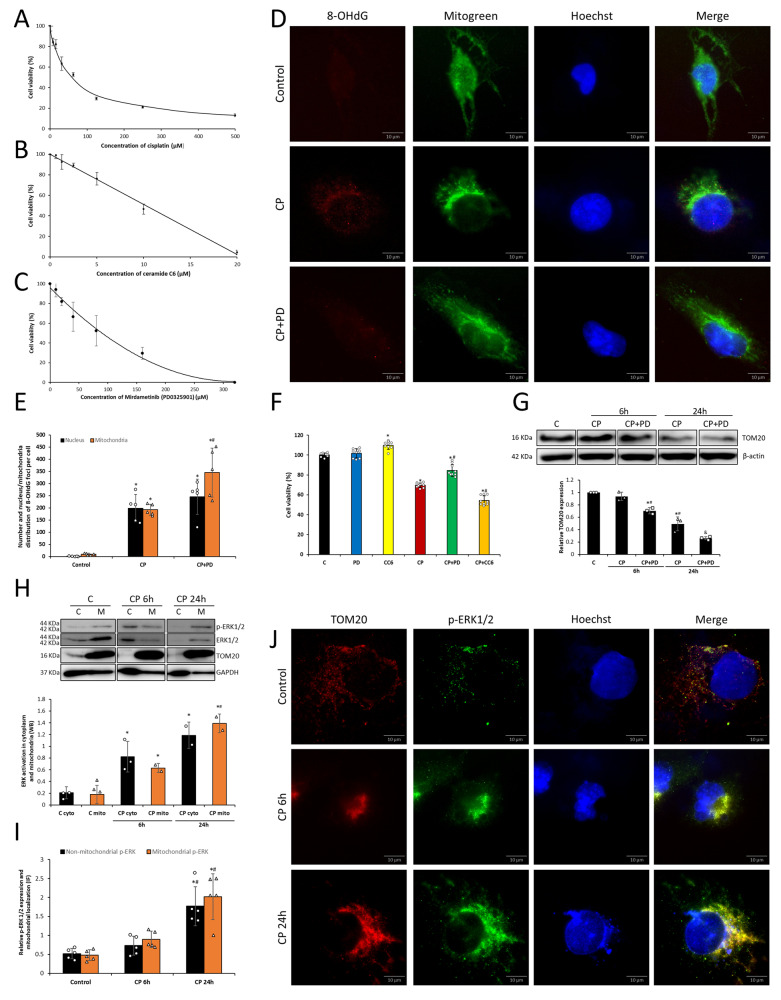
Localization and involvement of ERK in cell viability and mtDNA damage. The dose-dependent effect of cisplatin (CP) (**A**), mirdametinib (PD) (**B**), and ceramide C6 (CC6) (**C**) on HK-2 cell viability. CP 30 μM, PD 10 μM, and CC6 1 μM were selected for the experiments. The cells were treated for 24 h and cell viability was measured by the XTT assay (**F**). PD protected cells against CP-induced toxicity and ERK induction by CC6 decreased cell viability in cotreatment with CP. The percentage of cytotoxicity was calculated in comparison to untreated cells taken as 100%. Values are expressed as mean ± SD from three independent experiments performed in triplicates. Nuclear and mitochondrial damage amounts were determined with immunofluorescence analysis (IF) of 8-OHdG and nucleus/mitochondria colocalization **D** under 6 h treatment. CP induced both mitochondrial and nuclear DNA damage. PD had no effect on nuclear DNA damage but increased mitochondrial DNA damage compared to CP alone. 8-OHdG foci were measured in more than 30 cells (magnification 1000×) and quantified (**E**). Representative immunoblots of TOM20 and β-actin and graph bar of TOM20 normalized per β-actin (**G**). PD decreased mitochondrial number in both 6 h and 24 h treatments. Representative images of TOM20 (red), p-ERK (green), and nuclear stain Hoechst (blue) in HK-2 cells treated with vehicle control or with cisplatin (CP) for 6 and 24 h (**J**). IF analysis of p-ERK indicates that after the 6-h treatment activated ERKs are gathered around nuclei of the cells, and after the 24-h treatment, they are arranged in puncta. p-ERK distribution in the cells and its activation on mitochondria quantification of IF analysis (**I**) and calculation of its activation in raw cell mitochondrial and cytoplasmic fractions (**H**). *P < 0.05 vs control; ^#^*P* < 0.05 vs CP or CP 6 h, ^&^*P* < 0.05 vs CP 24 h. Data were analyzed by one-way ANOVA, followed by Tukey’s post-hoc test. The 50% inhibitory concentrations (IC_50_) were determined using linear and nonlinear regression analysis.

In 24-h treatments CP exhibited an IC_50_ value of 60.32 µM on HK-2 cells, PD exhibited an IC_50_ value of 89.42 µM and CC6 showed an IC_50_ value of 10.25 µM (Fig. 1A, B, and C). Doses of PD and CC6, used for further experiments did not affect cell viability compared to untreated cells (Fig. 1F). Inhibition of ERK before CP treatment increased cell viability compared to CP alone and further ERK activation during CP treatment decreased cell viability compared to CP alone in 24-h treatment, evidencing the role of ERK activation in the reduction of cell viability during CP treatment (Fig. 1F). Since CP treatment increases ERK activation, CC6 was not used in further experiments.

### ERK is involved in CP-induced mtDNA damage and maintenance of the mitochondrial population

Treatment with CP increased the number of 8-OHdG foci per cell in both mitochondria and nuclei (Fig. 1D, E). ERK inhibition resulted in no change in nDNA but an increase in the endogenous oxidative damage to mtDNA. TOM20 expression that correlates to the mitochondrial mass showed a significant decrease in the mitochondrial population during CP treatments as seen from relative TOM20 expression normalized to protein loading control β-actin (Fig. 1G, original blots for this Fig. and other Figs. are in Supplementary file). Inhibition of ERK activation in CP treatment resulted in a further decrease of TOM20 expression in both 6 and 24-h treatments (Fig. 1G), confirming the role of ERK activation in the preservation of the mitochondrial number/population.

### CP-induced ERK activation found in both cytoplasm and mitochondria

**Fig. 2:**
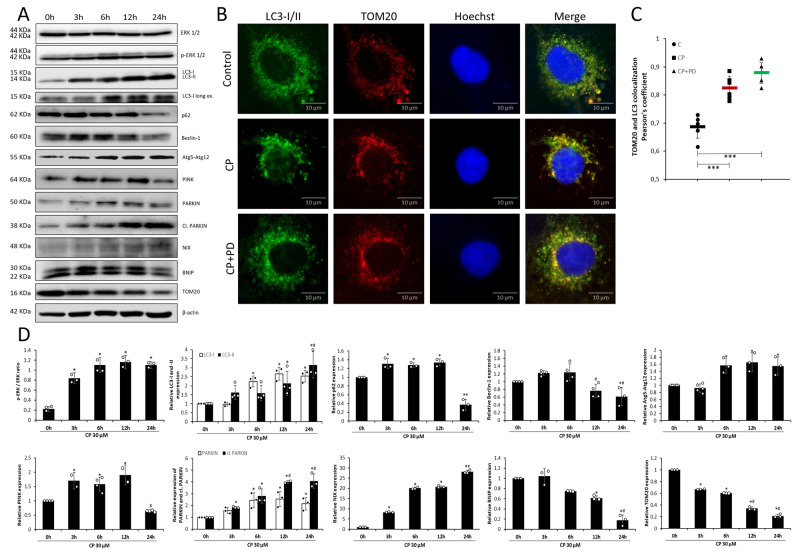
Autophagy and mitophagy in correlation to ERK. Representative immunoblots of ERK and phosphorylated ERK expression in HK-2 cells (**A**). Treatment with CP increased the ERK activation in the early phase (3 and 6 h) compared to control with a slight increase after 6-h treatment as seen from the quantification ratio of p-ERK/ERK (**D**). Representative immunoblots of LC3BI/II, p62, beclin-1, Atg5-Atg12, PINK1, PARKIN, cleaved PARKIN, NIX, BNIP, TOM20, and loading control protein (β-actin) expression in HK-2 cells in cisplatin (CP) treatment time points (**A**). The bar graphs demonstrate LC3-I and -II, p62, beclin-1, Atg-5-Atg12, PINK, PARKIN, and cleaved PARKIN, NIX, BNIP, and TOM20 expression, all normalized to β-actin (**D**) (*P < 0.05 vs control, ^#^*P* < 0.05 vs 6 h). Treatment with CP resulted in autophagy activation in HK-2 cells at 6–24 h compared to control. LC3-I/II levels increased gradually and were higher in later phases (12 and 24 h). Levels of p62 increased after 3 h-treatment and decreased after 24 h compared to control. Beclin-1 expression increased after 6 h and decreased after 12–24 h. Atg5-Atg12 complex formed at 6 h-treatment with no further increase. Treatment with CP resulted in mitophagy initiation in HK-2 cells in the early phase of CP treatment compared to control evidenced by protein expression change. Immunofluorescence analysis of LC3 and TOM colocalization in the early phase of treatment (6 h) (**B**) confirmed mitophagy activation that was not significantly affected during ERK inhibition (PD) in combination with CP treatment presented in differences between Pearson’s coefficient between groups (**C**) (***P < 0.001 vs control). Data was analyzed by one-way ANOVA followed by Tukey’s post-hoc test.

ERK is found to be phosphorylated promptly after the 3-h treatment with CP reaching its highest values after the 6-h treatment and remaining unchanged in later treatments seen from the p-ERK/ERK ratio (Fig. 2A, D). The localization of p-ERK has shown that the dispersed distribution in the cytoplasm changes drastically at the 6-h treatment with CP, towards the nucleus. Surprisingly, after the 24-h treatment, the localization of p-ERK was again found in dispersal distribution in the cytoplasm, this time formed in a more pronounced puncta (Fig. 1J). Quantification of p-ERK colocalizing with TOM20 showed that p-ERK seems to be equally distributed as “mitochondrial” and “non-mitochondrial” in control and CP-treated cells (Fig. 1I), which was confirmed by ERK activation (ratio of p-ERK and ERK) in raw cytoplasmic and mitochondrial cell fractions (Fig. 1H). Furthermore, the localization of mitochondria in cells during the early CP treatment suggests mito-nuclear communication, which, interestingly, strongly correlates with p-ERK localization (Fig. 1J).

### CP induces autophagy in the early stage of treatment which is more prominent in the late stage

LC3I/II showed increased expression of LC3-I, and a slight increase in LC3-II in the early stage but the expression of both proteins increased significantly in the late stage of CP treatments (Fig. 2A, D). p62 increased expression as early as the 3-h time point and maintained its levels until the 12-h time point, and at the 24-h time point showed significantly lower levels compared to all other time points (Fig. 2A, D). Beclin-1 levels increased in the early stage compared to the control and decreased at 12- and 24-h time points (Fig. 2A, D). Increased expression of Atg5-Atg12 was found at 6 to 24-h treatments compared to the control (Fig. 2A, D).

### Mitophagy induced by CP in the early stage of treatment has a protective role and ERK has no significant role in its conduction/occurrence

PINK1 expression increased in the early stage, while its levels significantly decreased at the late stage of CP treatment (Fig. 2A, D). PARKIN expression increased at the 6-h time point after which it was cleaved, evidenced by the increase in cl.PARKIN/PARKIN ratio at 12- and 24-h time points (Fig. 2A, D). CP-induced PINK1/Parkin mitophagy in the early stage and a shift towards apoptosis due to an increase of cleaved PARKIN levels. NIX expression increased in 3- to 12-h time points, with further increase in the late stage (Fig. 2A, D), pointing out possible induction of NIX-mediated mitophagy and autophagic cell death induction in the late stage. Levels of BNIP3, localized in the mitochondria, dropped in the late stage, and highly correlated with the changes in TOM20 expression in all time points (Fig. 2A, D). The mitophagy induction at the 6-h time point was confirmed with LC3I/II-TOM20 colocalization (Fig. 2B). During ERK inhibition no increase in Pearson’s correlation coefficient between CP + PD and CP alone groups occurred (Fig. 2C).

### ATP deprivation and AMPK/Akt crosstalk are emphasized only in the late stage of the CP treatment where ERK activation is responsible for the decrease in the reserve respiratory capacity

**Fig. 3:**
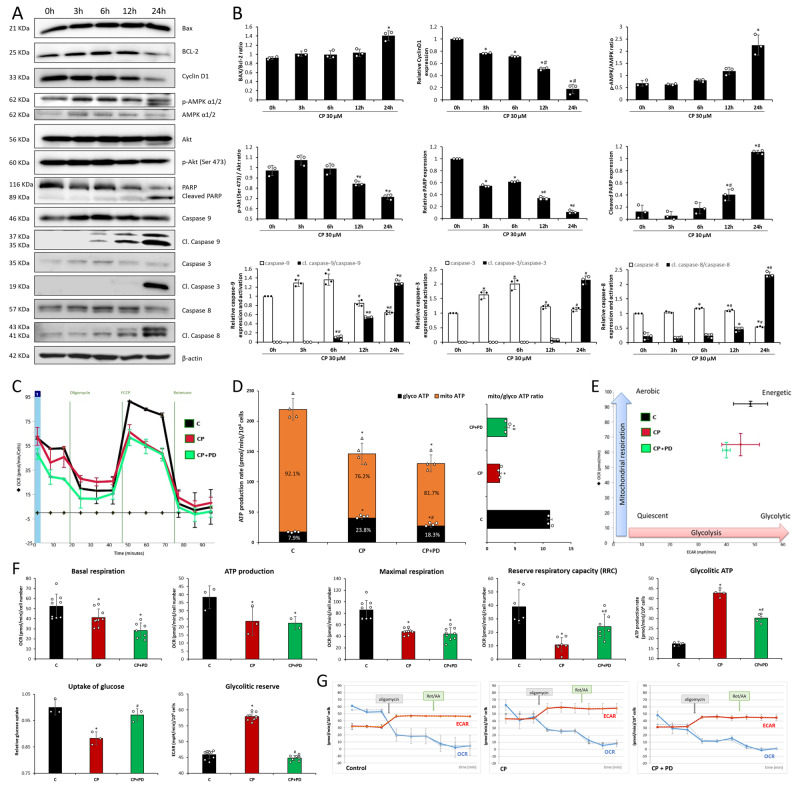
Apoptosis and cell cycle, ATP level, and glucose uptake in correlation to ERK. Representative immunoblots of Bax, Bcl-2, CyclinD1, p-AMPKα, AMPKα, AKT, p-Akt (Ser 473), PARP, cleaved PARP, caspase-9, cleaved caspase 9, caspase 3, cleaved caspase 3, caspase 8, cleaved caspase 8 and loading control protein (β-actin) expression in HK-2 cells in cisplatin treatment time points (**A**). The bar graphs demonstrate the Bax/Bcl-2 ratio, cyclin D1 expression, p-AMPKα/AMPKα ratio, p-Akt(Ser473)/Akt ratio, as well as the relative PARP expression, cleaved PARP expression, relative caspase-9, caspase 3 and caspase-8 expression (white bars) and activation (black bars) (**B**). All quantifications of expression were normalized to β-actin (*P < 0.05 vs control, #*P* < 0.05 vs to 6 h-treatment). Seahorse analysis of the effect of cisplatin (CP), ERK inhibitor (PD), and their combination (CP + PD) on mitochondrial function and cellular ATP gain in HK-2 cells. The ATP synthase inhibitor oligomycin, respiratory chain uncoupler FCCP, or complex I and III inhibitor rotenone/antimycin A were added to the culture medium as indicated (**C**). Basal respiration, ATP production, maximal respiration, and reserve respiratory capacity (**F**) were calculated from the oxygen consumption rate (OCR). ATP production rate in HK-2 cells was calculated based on the results of extracellular acid rate (ECAR) and OCR. The ATP production rate from glycolysis (glyco ATP) and mitochondrial oxidative phosphorylation (mito ATP), and their ratio (**D**) as well as the cell energy profile (**E**) are graphically presented. Glycolitic ATP production rate, determination of glucose uptake, and the glycolytic reserve estimated by ECAR in the presence of oligomycin (**F**). The changes in the OCR and ECAR after the addition of the oligomycin and rotenone/antimycin A to the culture media in the control cells, CP-treated, and in the presence of the combination of CP and PD (**G**). ECAR profiles were expressed as mpH/min/1.0 × 10^4^ cells. One-way ANOVA followed by Tukey’s posthoc test was used to analyze the data (mean ± SD). *P < 0.05 versus control, ^#^*P* < 0.05 versus the CP-treated group.

ATP deprivation can be evidenced by AMPK phosphorylation, as noticed here in the late stage of the CP treatment (Fig. 3A, B), due to impairment of mitochondrial function and number. It is interesting to note that in the late stage, Akt activation on Ser473, which is mainly due to ERK, decreases (Fig. 3A, B), in correlation with AMPK phosphorylation. Treatment with CP caused a decrease in basal respiration, maximal respiration, ATP production, and RRC (Fig. 3C, F). ERK inhibition during CP treatment only showed an increase in RRC, compared to CP (Fig. 3F).

### ERK activation plays a role in glucose uptake and glycolysis increase during CP treatment

CP treatment increased ECAR values compared to the control, which normalized when ERK activation was inhibited (Fig. 3F). CP increased the maximum rate of conversion of glucose to pyruvate or lactate which is probably due to a compensatory mechanism of decreased glucose uptake (Fig. 3E, F). Relative glucose uptake in cells decreased during CP treatment but ERK inhibition stabilized glucose consumption (Fig. 3F). CP decreases the mito/glyco ATP ratio that increased when ERK activation is inhibited (Fig. 3D).

### CP induces cell cycle inhibition in the early stage of the treatment with extrinsic and intrinsic apoptotic pathways induced mainly in the late stage

CyclinD1 decreased in the 3-h CP treatment, with further decrease and fading at 12- and 24-h treatments (Fig. 3A, B). Bax/Bcl-2 ratio increase occurred only in the 24-h treatment (Fig. 3A, B). Decrease in PARP expression (Fig. 3A, B) and its cleavage occurred at 12- (3-fold increase) and 24-h (tenfold increase) timepoint (Fig. 3A, B). Expression of full-length caspase-9 and increase in cleaved caspase-9/caspase-9 ratios increase drastically with a concomitant decrease in full-length caspase-9 expression in the late stage of treatment (Fig. 3A, B). Furthermore, the extrinsic pathway is activated only in the late stage evidenced by a 2.5-fold increase in cleaved caspase-8/caspase-8 ratios (Fig. 3A, B). The cleavage of caspase-3 increased in the late stage (Fig. 3A, B).

### ERK activation is required for full-length PARP expression/activation but does not affect cleaved PARP levels

**Fig. 4:**
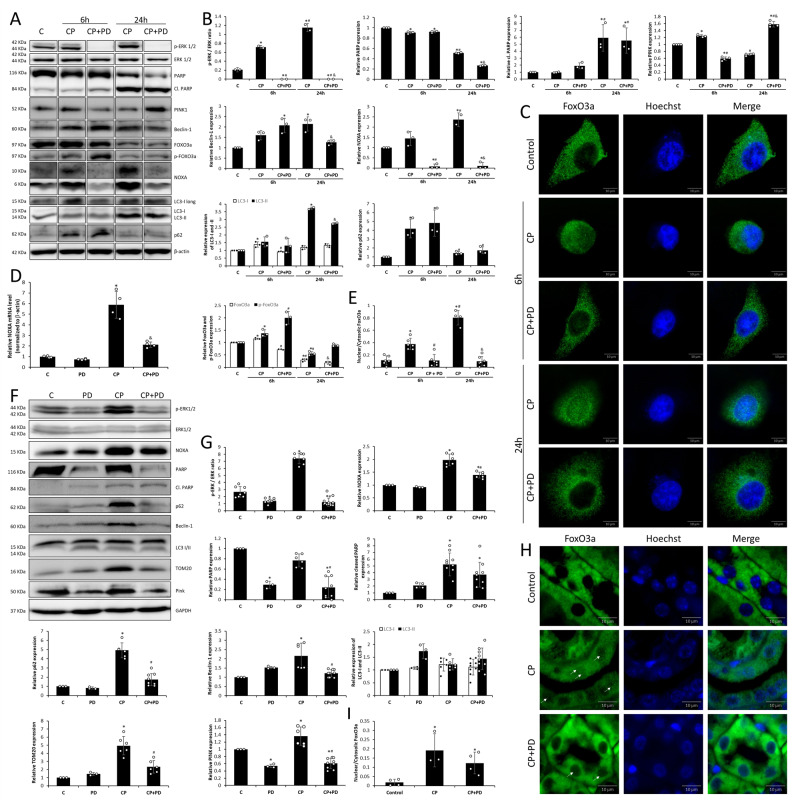
Impact of ERK on PARP, NOXA, and FoxO3a levels and mitophagy. Representative immunoblots of p-ERK, ERK, PARP, cl. PARP, PINK1, beclin-1, FoxO3a, p-FoxO3a (Ser294), NOXA, LC3-I/II, p62 and loading control protein (β-actin) expression in HK-2 cells in cisplatin (CP) and CP in combination with ERK inhibitor (PD) treatment at 6 and 24 h time points (**A**). The bar graphs demonstrate the p-ERK/ERK ratio, relative PARP, cl. PARP, PINK1, beclin-1, NOXA, LC3-I and LC3-II, p62 expressions all normalized to β-actin and FoxO3a and p-FoxO3a (Ser294) ratio (**B**). Nuclear translocation of FoxO3a under treatments and time points in HK-2 cells was determined with immunofluorescence analysis (**C**). FoxO3a intensity was measured in more than 30 cell nuclei and cytosol (magnification 1000×), quantified, and expressed as nuclear/cytosolic ratio (**E**). Real-time quantitative PCR relative expression levels of NOXA (normalized to β-actin) in HK-2 cells treated for 24 h, values represent averages of four biological replicates (**D**). Representative immunoblots of p-ERK, ERK, NOXA, PARP, cl. PARP, p62, beclin-1, LC3-I/II, TOM20, PINK, p62 and loading control protein (GAPDH) in BALB/c kidneys after cisplatin (CP), ERK inhibitor (PD) and their combination treatments (**F**). The bar graphs demonstrate the p-ERK/ERK ratio, relative NOXA, PARP, cl. PARP, p62, beclin-1, LC3-I and LC3-II, TOM20, PINK expressiona, all normalized to GAPDH (**G**). Nuclear translocation of FoxO3a in mouse kidneys was determined with immunofluorescence analysis (**H**). FoxO3a intensity was measured in more than ten random kidney areas in the nucleus and cytosol (magnification 1000×), quantified, and expressed as nuclear/cytosolic ratio (**I**). Data are presented as average ± SD and were analyzed by one-way ANOVA followed by Tukey’s posthoc test (*P < 0.05 vs control, ^#^*P* < 0.05 vs 6-h CP treatment (HK-2) or CP treatment (mice), ^&^*P* < 0.05 vs 24-h CP treatment (HK-2)).

ERK inhibition decreased full-length PARP expression at 24-h treatment compared to only CP treatment in vitro but had no significant effect on cleaved PARP expression (PARP inactivation) that results from caspase cleavage in the late stage (Fig. 4A, B), confirming the requirement of ERK activation for maximal PARP activation [16]. In vivo, decreased full-length PARP expression in treatment with PD alone, compared to the control, and no significant change in PARP cleavage (Fig. 4F, G). Treatment with CP, as expected, increased PARP cleavage with a concomitant decrease in full-length PARP (Fig. 4F, G). ERK inhibitor alone decreased PARP levels compared to the control (Fig. 4F, G). Inhibition of ERK activation during CP treatment showed decreased levels of full-length PARP compared to CP alone (Fig. 4F, G), correlating with in vitro experiments.

### ERK plays a role in mitophagy initiation in the early stage and autophagy induction in the late stage during CP treatment

ERK inhibition during CP treatment decreased PINK1 expression in the early stage (Fig. 4A, B) and, interestingly, increased the expression of PINK1 in the late stage (Fig. 4A, B). ERK inhibition did not affect Beclin-1 expression in the early stage but caused a further decrease in the late stage (Fig. 4A, B). ERK inhibition in the early stage decreased the increase in LC3-I by CP, and in the late stage decreased LC3-II levels (Fig. 4A, B). p62 expression levels were not interrupted by ERK inhibition (Fig. 4A, B). In vivo, CP increased the expression of p62, Beclin-1, PINK1, and TOM20, and when ERK is inhibited, their levels decrease (Fig. 4F, G). Interestingly, LC3-I/II levels did not significantly change, where inhibition of ERK does not seem to have a role (Fig. 4F, G), resembling the in vitro early phase.

### ERK activation is required for NOXA expression during CP treatment

The expression of NOXA increased in early and late CP treatment, and its expression was found fully blocked when ERK activation was inhibited (Fig. 4A, B). mRNA expression of NOXA showed that in the late stage, CP causes upregulation of the NOXA gene, which decreases with the application of the ERK inhibitor (Fig. 4D). Also, NOXA expression is found to increase under CP treatment and is dependent on ERK activation in vivo (Fig. 4F, G). Although ERK activation is not fully inhibited in the mouse model as seen in the in vitro experiments, the decrease of NOXA expression correlates with ERK phosphorylation inhibition.

### ERK activation is required for FoxO3a nuclear translocation, but not phosphorylation of FoxO3a on Ser294 during CP treatment

FoxO3a expression increased in the early stage of treatment and normalized when ERK was inhibited (Fig. 4A, B). Phosphorylation of FoxO3a on Ser294 and its degradation increased when ERK activation was inhibited (Fig. 4A, B). In the late stage of the treatment, FoxO3a expression decreased due to increased phosphorylation in the early stage and degradation and remained unchanged when ERK activation was inhibited (Fig. 4A, B). Under CP treatment, FoxO3a enters the nucleus in the early stage, and especially in the late stage mediated by ERK activation, shown by the decrease in nuclear translocation of FoxO3a when ERK phosphorylation is inhibited (Fig. 4C, E). Similar results were obtained in the in vivo model where ERK inhibition was not full enough to significantly block the nuclear translocation of FoxO3a (Fig. 4H, I).

## Discussion

Combining ERK inhibitors with chemotherapies is a potential clinical approach in cancer treatment [17] and has not been investigated in the attenuation of CP-induced AKI. The literature presents conflicting results and different roles of ERK in the regulation of autophagy and apoptosis in different experimental models with different degrees of injury, where ERK activation and its effects on the cells seem to depend on multiple factors, including the degree of damage. ERK signaling can mediate apoptosis, autophagy, and senescence depending on cell type and stimulus [18]. As well as suppressing pro-apoptotic proteins, ERK1/2 can enhance the activity of anti-apoptotic molecules to promote cell survival or regulate the activity of anti- and pro-apoptotic transcription factors [19]. On the other hand, CP induces apoptosis through the ERK pathway in renal proximal tubule cells [20], and ERK inhibition can prevent cell death caused by CP [21]. Regarding neuronal apoptosis, ERK has a dual role due to the different kinds of neurons studied [22]. Also, ERK activation can facilitate the apoptosis induced by CP in HeLa cells [23], or mediate anti-apoptotic effects in human embryonic fibroblast in apoptosis induced by mild DNA damage [24]. Depending on the duration, the magnitude, and its subcellular localization, ERK activation controls different cell responses [25]. It is imperative to control the spatiotemporal activity of the ERK signaling pathway to achieve exquisite control over almost all cell functions [18]. The specificity of activation or inhibition of downstream effectors determines the consequence of ERK1/2 activation on cell survival, which is anti-apoptotic or in some cases, pro-apoptotic [19]. We hypothesized that in the early stage of CP injury, cells activate protective mechanisms, and in the late stage, are leaning towards death where ERK activation will contribute differently to mechanisms. Our in vitro studies show that ERK activation is responsible for overall cell viability in kidneys during CP injury. To clarify the role of ERK in stages of CP injury we defined the timeline of events since ERK is phosphorylated in both. When the activatory domain of ERK is phosphorylated, it not only activates the kinase but also alters its conformation, releasing ERKs from their anchoring proteins [26] and moving to the nucleus, mitochondria, and cytoplasm [5, 27] to regulate specific activities [28, 29]. p-ERK is found to be equally distributed as mitochondrial and non-mitochondrial despite CP treatment. In the early stage of CP treatment, p-ERK targets the nucleus, as a transcription factor, coinciding with the movement of mitochondria, and crosstalk between mitochondria and the nucleus is necessary to create a well-functioning mitochondrial population [30]. p-ERK has a role in the maintenance of mitochondrial populations during CP injury. In the in vitro model, the mitochondrial population remained unchanged in early and decreased in the late stage. On the other hand, an increase in the mitochondrial number during CP-induced injury in vivo was observed, which points out possible enhanced mitochondrial biogenesis or due to increased energy demand that originated from mitochondrial damage, since it more resembles the early phase in vitro. Interestingly, indicative mtDNA lesions, caused by CP are increased by p-ERK. Possibly due to the protective role of p-ERK to induce mitophagy in the early stage, evidenced also by an increase in ERK-mediated PINK1 levels in the early stage, and initiation of mitophagy during CP-injury, still, p-ERK had no impact on its conduction. PINK1/PARKIN mitophagy occurs in the early stage of CP treatment as protective when ATP is not yet deprived, confirmed with p62 required for mitophagy, after which PARKIN cleaves and causes a shift toward apoptosis. PARKIN is cleaved during apoptosis by caspase-mediated proteolysis [31] but also regulates mitophagy and apoptosis [32]. Through direct inhibition of Bcl-2 homologous antagonist/killer (BAK), PARKIN suppresses errant apoptosis, allowing the effective clearance of damaged mitochondria [33]. The fact that apoptosis induced by CP can be suppressed by stimulating mitophagy [34] agrees with our results. Previously, NIX was shown to be upregulated, while PINK1 and Parkin were downregulated by CP treatment [35] which happened in our experiments in the late phase, indicating an occurrence of NIX-mediated autophagy together with general autophagy, with p62 levels that decreased, and increased Atg5-12 confirming strong general autophagy activation. ERK activation by CP is responsible for autophagy in the late stage which is induced as a cell’s response to excessive damage and ATP deprivation, since mitochondrial stress is tolerated by the cells through mitophagy until the damage becomes extensive, causing the cells to undergo apoptosis [36]. When intracellular ATP levels reduce, AMPK activates, by phosphorylation [37], as noticed in the late stage of CP treatment, which coincided with TOM20 decrease and correlated with autophagy. Activated AMPK inhibits the activity of Akt by dephosphorylating Ser473 [38] which agrees with our results in the late stage where Akt activation on Ser473, mainly due to ERK, decreases. It is also possible for AMPK and Akt to regulate each other’s phosphorylation [39], which seems to be the case in the late stage of CP treatment. Mitochondrial Bcl-2’s anti-apoptotic function can be decreased when ERK phosphorylates it [40]. It was found that damaged DNA activates PARP through phosphorylation by ERK under conditions promoting cell death [41], such as the late stage described in this paper. ERK activation by CP correlates with full-length PARP expression in the late stage of injury. Having PARP knocked out genetically or pharmacologically significantly reduces CP-induced renal dysfunction and histopathological damage [42]. The cleavage of PARP by caspase-3, which inactivates it and inhibits PARP’s DNA-repairing abilities, was unaffected by ERK activation inhibition at the late stage of CP injury. Also, PARP activation via direct interaction with p-ERK2 has been described, which does not involve binding to DNA and is unrelated to DNA damage [43]. PARP can over-activate, resulting in ATP depletion and cell death [44]. The cell operates on a fraction of its mitochondrial bioenergetic capacity under normal/unstressed conditions, with the RRC referring to the difference between the maximum and basal respiratory capacities [45]. ERK activation results in a decrease in RRC during the late stage. RRC can increase supply when energy demand exceeds supply, preventing an ATP crisis [45], which happened in ERK inhibition during CP injury. Based on our viability results during the inhibition of ERK activation in CP treatment agree with the fact that RRC correlates with improved cell survival [46]. Damage to mitochondria caused by CP alters the levels of glycolysis-related metabolites [47]. Glycolysis can be used as a compensatory pathway to produce ATP in AKI [48]. ERKs are known to regulate the redirection of energy harvest to glycolysis in malignant and highly proliferating cells through the regulation of key metabolic enzymes [49]. ERK activation seems to inhibit cells’ ability to uptake glucose in cells during CP treatment. As a result of nutrient starvation, ERK can also drive the expression of the prodeath protein NOXA that controls autophagy or apoptosis decisions [50]. Since NOXA is generally considered a pro-apoptotic protein, blocking its induction suppresses apoptosis [51]. Furthermore, it has been shown that ERK activation promotes autophagy through NOXA expression [52]. We found that ERK activation is required for NOXA both gene and protein expression during CP treatment and therefore seems that inhibition of ERK activation and shift in glucose metabolism alleviate the amount of apoptosis and autophagy. CP’s cytotoxic effects are also mediated by FoxO3a [53, 54] and for its transcriptional activity, FoxO3a is shuttled from the cytoplasm to the nucleus or mitochondria [55]. ERK activation is responsible for FoxO3a nuclear translocation and subsequent transcriptional activity during CP injury, possibly also for NOXA. ERK can directly phosphorylate FoxO3a, which is then degraded [56] but we found that during CP treatment, ERK activation is not responsible for the phosphorylation of FoxO3a on Ser294, which can be due to phosphorylation on Ser294 by other MAPKs, such as JNK and p38 [57].

In summary, ERK activation is a crucial factor in CP-induced kidney injury that impacts multiple proteins that can decide between survival and cell death. Also, the ERK signaling pathway has been identified as a promising therapeutic target for cancer therapy [58]. Several different mutations, involving BRAF or NRAS, exert an oncogenic effect by activating the MAPK pathway, increasing cellular proliferation [59]. Mirdametinib at clinically relevant doses suppresses bladder tumor growth in patient-derived xenograft models [60]. MEK inhibitor combination therapies can help overcome CP resistance [61]. Thus, ERK inhibition should not deploy additional problems such as interference with chemotherapy while mitigating nephrotoxicity, and should enable a new approach to nephrotoxicity prevention and application in clinics resulting in high-efficacy CP chemotherapy.

## Methods

### Cell culture

Human renal proximal tubular cells (HK-2), obtained from American Type Culture Collection (ATCC®, CRL-2190™, Rockville, MD, USA) were grown in low glucose Dulbecco’s Modified Eagle’s Medium (DMEM) (D6046, Sigma-Aldrich, Steinheim, Germany) supplemented with penicillin/streptomycin (PS-B), and 10% fetal bovine serum (FBS-HI-11B) (all from Capricorn Scientific, Ebsdorfergrund, Germany) at 37 °C in 5% CO_2_ in a humidified atmosphere incubator (BS-010425-A01, S-Bt Smart, Biotherm, Biosan, Riga, Latvia) in tissue culture flasks (90026 or 90076, TPP, Trasadingen, Switzerland). Before each treatment, cells were collected by trypsin-ethylenediaminetetraacetic acid (0.05%) in Dulbecco’s phosphate-buffered saline (DPBS) (TRY-1B, Capricorn Scientific, Ebsdorfergrund, Germany), counted and cultured until 80% confluence.

### In vitro experimental design

Treatments are divided into the early and late stages of treatment. The early stage represents the initial events (3, 6, or 12 h treatment) before apoptosis events in the cell that occur in the late stage (24 h treatment). Right before treatments, a 10 mM fresh stock solution of cisplatin (CP) (P4394, 99.99%) in dimethyl sulfoxide (DMSO) (41640) (both from Sigma-Aldrich, Steinheim, Germany) was prepared. To reach final concentrations, 10 mM stock solutions of Mirdametinib (MEK inhibitor, PD035901) (PD) (S1036, 99.96%) (Selleck Chemicals, Huston, TX, USA) and 1 mM stock solution of C6 ceramide (ERK activator) (CC6) (d18:1/6:0) (860506P, >99%) (Avanti Polar Lipids, Birmingham, AL, USA) in DMSO were added to complete medium 2 h before treatment with CP. After reaching 80% confluence on tissue culture flasks or 96 and 24-well plates (Z707902 and Z707791, TPP, Trasadingen, Switzerland), cells were treated with prepared solutions and collected at different time points after treatment (0, 3, 6, 12, and 24 h) for further analysis. In all further experiments, a CP dose of 30 µM was used. For the ERK inhibitor and activator, final doses of 10 µM and 1 µM were used, respectively. The final concentration of DMSO in the complete medium was <0.5%. All experiments were performed three times in triplicates.

### Cell viability assay

To access the values of half-maximal inhibitory concentration (IC_50_) of tested chemicals and get an insight into the viability of the cells in combinatory therapy, the XTT Cell Viability Assay Kit (#9095 S, Cell Signaling Technologies, Beverly, MA, USA) was used. This colorimetric assay shows the metabolic capability of the cells to reduce XTT into a colored product measured at 450 nm. Cells were seeded at 96 well plates, treated and absorbance was measured after 24 h using a microplate reader (BIOT-808IU, BioTek Elx808, Winooski, VT, USA).

### Western blot analysis

After treatments, cells were collected by scraping, and kidneys were homogenized (POLYTRON PT-MR 1600E, Kinematica AG, Malters, Switzerland) and whole cell protein content was obtained using radioimmunoprecipitation buffer with protease inhibitors (sc-24948, Santa Cruz Biotechnology, Dallas, TX, USA) with the addition of phosphatase inhibitor PhosSTOP (04-906-845-001, Roche Diagnostics GmbH, Mannheim, Germany). Cytosolic and mitochondrial protein fractions were obtained using a cell fractionation kit (AB65320, Abcam, Cambridge, UK). Protein concentration in samples was determined using Pierce™ Rapid Gold BCA Protein Assay Kit (1863381, Thermo Scientific™, Waltham, MA, USA). Equivalents of 10, 20, or 30 µg of proteins were separated by 8%, 10%, 12.5%, or gradient sodium dodecyl sulfate (SDS)-polyacrylamide gel electrophoresis using Mini-PROTEAN® Electrophoresis System (1658002FC, Bio-Rad Laboratories, Hercules, CA, USA) and transferred onto Immobilon® Polyvinylidene fluoride (PVDF) membrane (EVH00005, Merck-Millipore, Burlington, MA, USA) using Trans-Blot® SD System (1703848, Bio-Rad Laboratories, Hercules, CA, USA). After blocking with 5% non-fat dry milk (#170-6404, Bio-Rad Laboratories, Hercules, CA, USA) in Tris-buffered saline (TBS) (T6066, Sigma-Aldrich, Steinheim, Germany) (TBST) 0.01 M, pH 7.4 containing 0.1% Tween-20 (17-1316-01, Amersham Biosciences, Amersham, UK), membranes were incubated at 4 °C overnight or for 1 h at room temperature with primary antibodies against ERK1/2 (#4695, 1:1000), p-ERK1/2 (Thr202/Tyr204) (#4370, 1:1000), Microtubule-associated protein 1 A/1B-light chain 3 (LC3BI/II) (#2775, 1:1000), Beclin-1 (#3495), Autophagy Related 5 (ATG5) (#12994, 1:1000), PTEN-induced kinase 1 (PINK1) (#6946, 1:1000), PARKIN (#2132, 1:1000), AMP-activated protein kinase (AMPK) (#2532, 1:1000), p-AMPK (Thr172) (#2535, 1:1000), Protein kinase B (AKT) (#9272, 1:1000), p-AKT (Ser473) (#4060, 1:2000), poly(ADP-ribose) polymerase (PARP) (#9542, 1:500), Caspase-3 (#9661, 1:1000), Caspase-8 (#4790, 1:1000), cleaved Caspase-8 (#9496, 1:1000), Forkhead box protein O3a (FoxO3a) (#2497, 1:1000), p-FoxO3a (Ser294) (#5538, 1:1000), The sequestosome-1 (p62) (#5114, 1:1000) (all from Cell Signaling Technologies, Beverly, MA, USA), Translocase of outer membrane 20 (TOM20) (sc-136211, 1:500), BNIP3-like (BNIP3L/NIX) (sc-166332, 1:500), BCL2/adenovirus E1B 19kD protein-interacting protein 3 (BNIP) (sc-56167, 1:500), Phorbol-12-myristate-13-acetate-induced protein 1 (NOXA) (sc-56169, 1:500) (Santa Cruz Biotechnology, Dallas, TX, USA), Bcl-2-associated X protein (BAX) (ab32503, 1:1000), B-cell lymphoma 2 (BCL-2) (ab7973, 1:100), Cyclin D1 (ab16663, 1:100), Caspase-9 (ab185719, 1:1000), β-actin (ab8226, 1:10000) (all from Abcam, Cambridge, UK) and Glyceraldehyde-3-phosphate dehydrogenase (GAPDH) (HRP-60004, 1:20000, Proteintech, Rosemont, IL, USA). The membranes were washed in TBST and incubated with appropriate HRP-linked secondary antibody, rabbit IgG (#7074, 1:20000, Cell Signaling Technologies, Beverly, MA, USA), mouse IgG (ab97023, 1:40000, Abcam, Cambridge, UK) or mouse IgGκ (sc-516102, 1:3000, Santa Cruz Biotechnology, Dallas, TX, USA). After washing in TBST, signals were obtained using SignalFire Elite ECL Reagent (12757P, Cell Signaling, Danvers, MA, USA) and ImageQuant™ LAS 4000 mini (28-9558-10, GE Healthcare, Chicago, IL, USA). The intensity of the bands was assayed by image analysis software ImageJ version 1.53n (Bethesda, MD, USA). For ERK activation in raw cell fractions, a correction factor for mitochondrial/cytosolic debris in fractions was used for quantification.

### RNA extraction and quantitative real-time PCR

Total RNA was extracted from HK-2 cells harvested after 24 h with TRI Reagent solution (AM9738, Ambion, TX, USA). The concentration of the isolated RNA was measured using a NanoVue spectrophotometer (28923216, GE HealthCare, Chicago, IL, USA), and quality was determined using the A260/A280 and A260/A230 ratios. Complementary DNA was synthesized from 5 μg total RNA according to the High-Capacity cDNA Reverse Transcription Kit (4368814, Applied Biosystems, CA, USA) guidelines.

Primer sequences for NOXA were taken from the Harvard PrimerBank (PrimerBank ID 21595743a1) and ordered from Thermo Scientific™ (Waltham, MA, USA (LSG, Bioproduction)). The forward sequence was 5′-ACCAAGCCGGATTTGCGATT-3′ and the reverse was 5′-ACTTGCACTTGTTCCTCGTGG-3′. β-actin primer sequences (forward 5′-AGAAAATCTGGCACCACACC-3′ and reverse 5′-GGGGTGTTGAAGGTCTCAAA-3’) were taken from previously published research [62] and ordered from Metabion (Planegg, Germany). For quantitative real-time polymerase chain reaction (qPCR) analysis, 0.1 μg of cDNA was subjected to PCR amplification with the use of Power SYBR Green PCR Master Mix (4367659, Applied Biosystems, CA, USA) on a 7300 Real-Time PCR System (4351103, Applied Biosystems). All RT-qPCR reactions were run in duplicates, and a no-reverse transcriptase control was used for each run to monitor genomic DNA contamination. mRNA NOXA expression levels were normalized to β-actin.

### Immunofluorescence

After treatments on round microscopic slides (9161064, Thermo Scientific™, Waltham, MA, USA) in 24-well plates, cells were fixed in methanol (UN1230, T.T.T., Sveta Nedjelja, Croatia) or paraformaldehyde (FNB4, Biognost, Zagreb, Croatia), washed, and blocked in 5% bovine serum albumin fraction V (BSA) (A3059, Sigma-Aldrich, Steinheim, Germany) for 2 h. Immunofluorescence analysis of mice kidneys was performed on 4 µM paraffin sections that were deparaffinized and rehydrated with xylene (BC, Biognost, Zagreb, Croatia) and ethanol (P147303, GRAM-MOL, Zagreb, Croatia) solutions, respectively. Antigen retrieval was performed for 20 min at 95 °C in citrate buffer solution (10 mM, 0.05% Tween20, pH 6.0) (106448, Merck-Millipore, Burlington, MA, USA), and slides were blocked in 3% BSA for 2 h. Slides were then incubated with primary antibodies against 8-hydroxy-2-deoxyguanosine (8-OHdG) (sc-66036, 1:200, Santa Cruz Biotechnology, Dallas, TX, USA), p-ERK1/2 (#4370, 1:200), LC3-I/II (#2775, 1:200), TOM20 (sc-136211, 1:500), or FoxO3a (#2497, 1:200) at 4 °C overnight. The slides were then washed and incubated with goat anti-rabbit IgG H&L (Alexa Fluor® 488) (ab150077, 1:500, Abcam, Cambridge, UK) or mouse-IgGκ BP-CFL 594 (sc-516178, 1:200, Santa Cruz Biotechnology, Dallas, TX, USA) secondary antibodies for 1 h at room temperature and counterstained with nuclear stain Hoechst 33342 (10 mg/ml) (B2261, Sigma-Aldrich, Steinheim, Germany) for 1 min (cells) or 15 min (tissue), washed and embedded in the mounting medium Mowiol 4-88 (0713.2, Carl Roth, Karlsruhe, Germany) with DABCO (D27802, Sigma-Aldrich, Steinheim, Germany). Before the fixation, cells were stained with Mitotracker™ green (200 nM) (y7530, Thermo Scientific™, Waltham, MA, USA) for 20 min, for determination of mitochondrial colocalization with 8-OHdG. Digital images were obtained using a fluorescence microscope (IX73, Olympus, Tokyo, Japan) with a magnification of 1000×. Images were then analyzed using the image analysis software ImageJ in more than 50 cells for each group.

### Seahorse analysis

The cellular Oxygen Consumption Rate (OCR) and Extracellular Acidification Rate (ECAR) were measured in real-time with an XFe24 Extracellular Flux Analyzer and Seahorse XF Cell Mito Stress Test (103015-100, Seahorse Agilent Technologies, Inc., USA) following manufacturer’s instructions (all other parts needed for Seahorse analysis were from Seahorse Agilent Technologies, Inc., USA as recommended). The cellular rate of ATP production, provided by glycolysis and mitochondrial oxidative phosphorylation, directly reflects cellular energetic demands to fulfill all cellular functions and is adaptable according to changes in the environment. Oligomycin inhibits ATP synthase linked to mitochondrial ATP production. Carbonyl cyanide 4-(trifluoromethoxy)phenylhydrazone (FCCP) is an agent that inhibits the proton gradient and disrupts the mitochondrial membrane potential, and inhibitors rotenone and antimycin A shut down mitochondrial respiration and enable the calculation of non-mitochondrial respiration. OCR measured under basal conditions is basal respiration, but influenced by several before-mentioned uncouplers allows the tracking of maximal respiration, total ATP production, and reserve respiratory capacity (RRC). Briefly, cells were seeded in 24-well plates at a density of 1.5 × 10^4^ cells/well for 24 h and then treated with CP, PD, and their combination for another 24 h. On the day of the assay, the medium was removed from the wells, and assay medium (XF-Base medium with 10 mM glucose, 1 mM pyruvate, and 2 mM L-glutamine) was added into the wells and incubated at 37 °C for 1 h in the non-CO_2_ atmosphere. The injection ports of the sensor cartridge were filled with mitochondrial inhibitors oligomycin (port A), FCCP (carbonyl cyanide-4 (trifluoromethoxy) phenylhydrazone, port B), and rotenone/antimycin A (port C), with the final well concentration of 1.5 μM, 1 μM, and 0.5 μM, respectively. The measurement cycle consisted of a 3-min mix, a 2-min wait, and a 3-min measurement. Three to four wells were used for each experimental group. Basal OCR represents the rate of mitochondrial respiration of the cells and is normalized to the final cell number, calculated from viability assay for each group. Mitochondrial functional parameters (basal respiration, ATP production, maximal respiration, and spare respiratory capacity) were calculated based on OCR measurements using mitochondrial inhibitors as modulators (supported by Report Generator User Guide, Seahorse Agilent Technologies, Inc., USA). All calculations necessary to convert OCR and ECAR to units of ATP production rate were performed according to Agilent’s instructions of “Quantifying Cellular ATP Production Rate Using Agilent Seahorse XF Technology”. The total ATP production rate is the sum of the ATP production from mitochondrial oxidative phosphorylation (mito ATP) and glycolysis (glyco ATP). The glycolytic reserve for differently treated cells was estimated by determining the ECAR in the presence of oligomycin. ECAR profiles were expressed as mpH/min/1.0 × 10^4^ cells.

### Glucose quantification in cell media

Determination of glucose concentration in the media after treatments was conducted by Glucose GOD FS* reagent for quantitative in vitro determination of glucose (125000010023, DiaSys Diagnostic Systems, Holzheim, Germany). Shortly, after 24-h treatments, cell media was collected, and incubated with glucose oxidase and peroxidase for 20 min at room temperature and the absorbance was measured at 500 nm on S-200 Spectrophotometer (BOE 8620000, Boeco, Hamburg, Germany). The concentration of glucose and its uptake by cells was calculated according to glucose standards and cell media.

### In vivo experimental design

Male, eight to ten-week-old BALB/cN mice were used in the experiment and were purchased from the Central Animal Facility of the Faculty of Medicine, University of Rijeka, Croatia. All mice were housed in plastic cages and maintained under controlled environmental conditions: 12-h light/dark cycles, constant temperature of 20 ± 1 °C, and humidity of 50 ± 5%. They were fed with standard pellet food (4RF21GLP, Muceola, Milano, Italy) and had free access to tap water. Before conducting the experiments, we calculated the necessary sample size and while conducting experiments and assessing the results, the investigators were not blinded to allocation. For the experiment, mice were randomly divided into experimental groups, each consisting of 6−8 mice. The control group (group I, *n* = 6) received saline by oral gavage. Group II received a single intraperitoneal injection of CP (13 mg/kg, *n* = 6). The dose of CP was determined in previous experiments [63]. Group III received PD0325901 (3 mg/kg, orally, *n* = 8), and group IV was injected with CP and oral gavage of PD0325901, respectively, for two consecutive days, 48 h after CP administration. The PD0325901 dose was selected and administered orally like in our previous experiments [64]. On the fifth day, mice were euthanized and kidneys were collected, one was immersed in buffered 4% paraformaldehyde solution for 48 h to obtain histological sections and one kidney was used for Western blot analyses.

### Statistical analysis

The IC_50_ values were determined using linear and non-linear regression analysis. Statistical differences between group means were determined by one‐way analysis of variance (ANOVA). Tukey’s post hoc test was used to compare multiple groups. Analysis was done with StatSoft STATISTICA 13 (StatSoft Inc., Tulsa, USA). All quantitative data are expressed as mean ± standard deviation. Differences with *P* < 0.05 were considered statistically significant (*P < 0.05 vs control, ^#^*P* < 0.05 vs 6-h CP treatment (in vitro) or CP treatment (in vivo), ^&^*P* < 0.05 vs 24-h CP treatment (in vitro), ***P < 0.001 vs control).

### Ethics statement

All experimental procedures were carried out according to the Croatian Animal Care and Ethics legislation and were approved by the local governmental authorities (i.e., Ministry of Agriculture, Veterinary and Food Safety, permit no. HR-POK-024).

### Supplementary information

Supplementary material

## Data Availability

The raw data supporting the conclusions of this article will be made available by the authors, without undue reservation, to any qualified researcher.
