# Supplementary material for: The interplay of mitophagy, autophagy, and apoptosis in cisplatin-induced kidney injury: involvement of ERK signaling pathway
Source: Cell Death Discov. 2024 Feb 24;10:98. doi: 10.1038/s41420-024-01872-0 (PMC10894217; doi:10.1038/s41420-024-01872-0)
Supplement: Supplementary file 1 — Supplementary material [file 41420_2024_1872_MOESM1_ESM.pdf]

Left: Chemiluminescence, Right: Digitalization at the same position

Figure 1G:

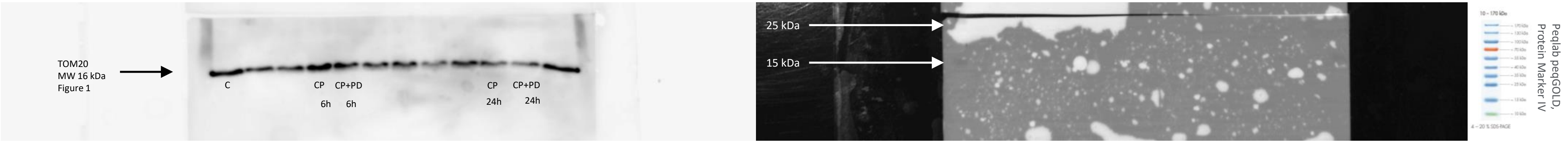

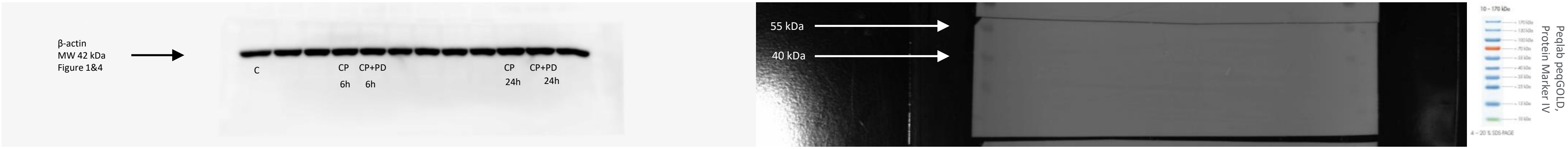

Figure 2A:

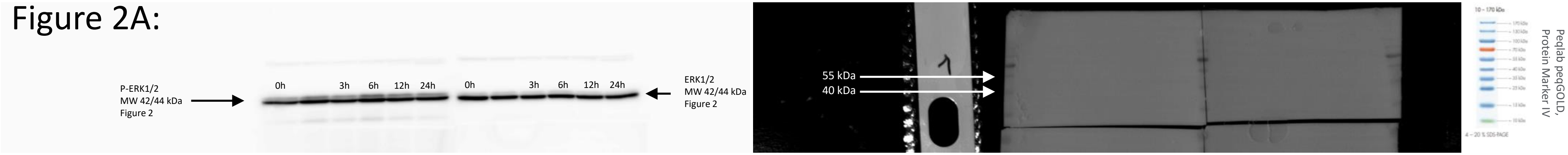

Figure 2A:

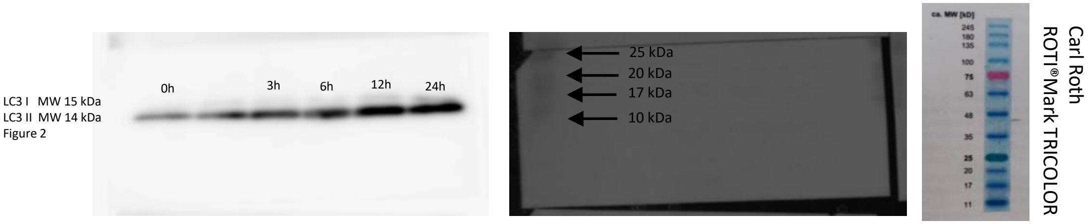

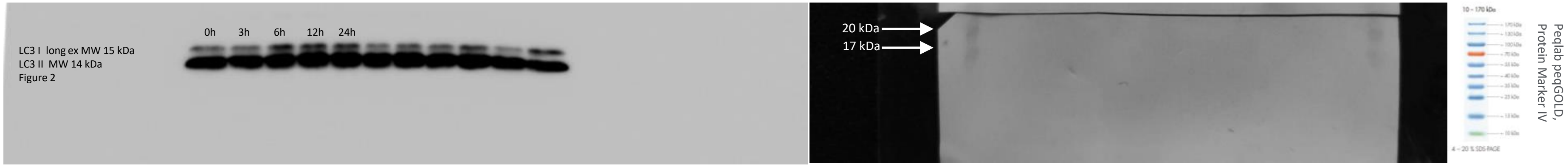

Figure 2A:

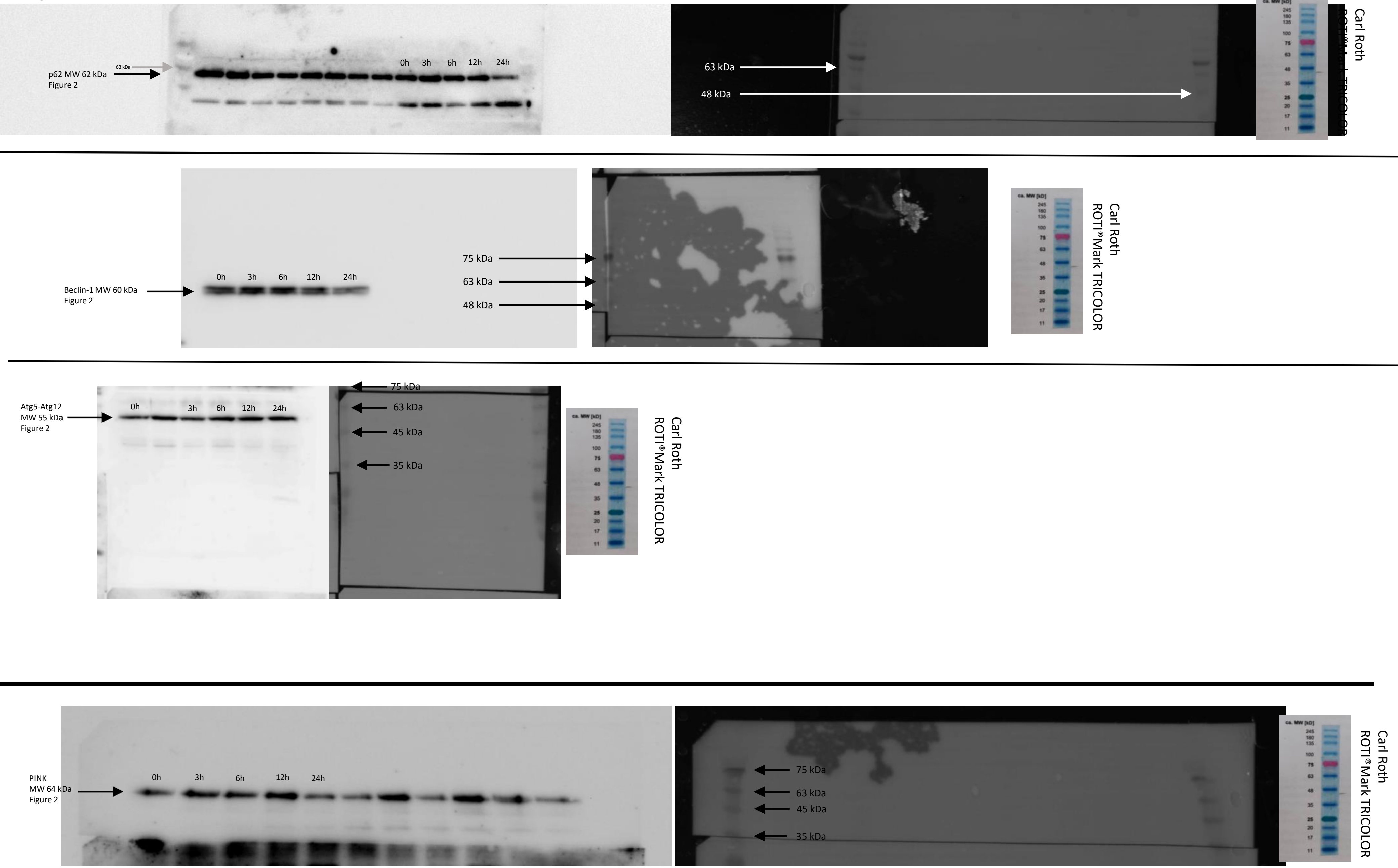
p62 MW 62 kDa
Figure 2
63 kDa
0h 3h 6h 12h 24h
63 kDa
48 kDa
Carl Roth
Beclin-1 MW 60 kDa
Figure 2
0h 3h 6h 12h 24h
75 kDa
63 kDa
48 kDa
Carl Roth
ROTI®Mark TRICOLOR
Atg5-Atg12
MW 55 kDa
Figure 2
0h 3h 6h 12h 24h
75 kDa
63 kDa
45 kDa
35 kDa
Carl Roth
ROTI®Mark TRICOLOR
PINK
MW 64 kDa
Figure 2
0h 3h 6h 12h 24h
75 kDa
63 kDa
45 kDa
35 kDa
Carl Roth
ROTI®Mark TRICOLOR

Figure 2A:

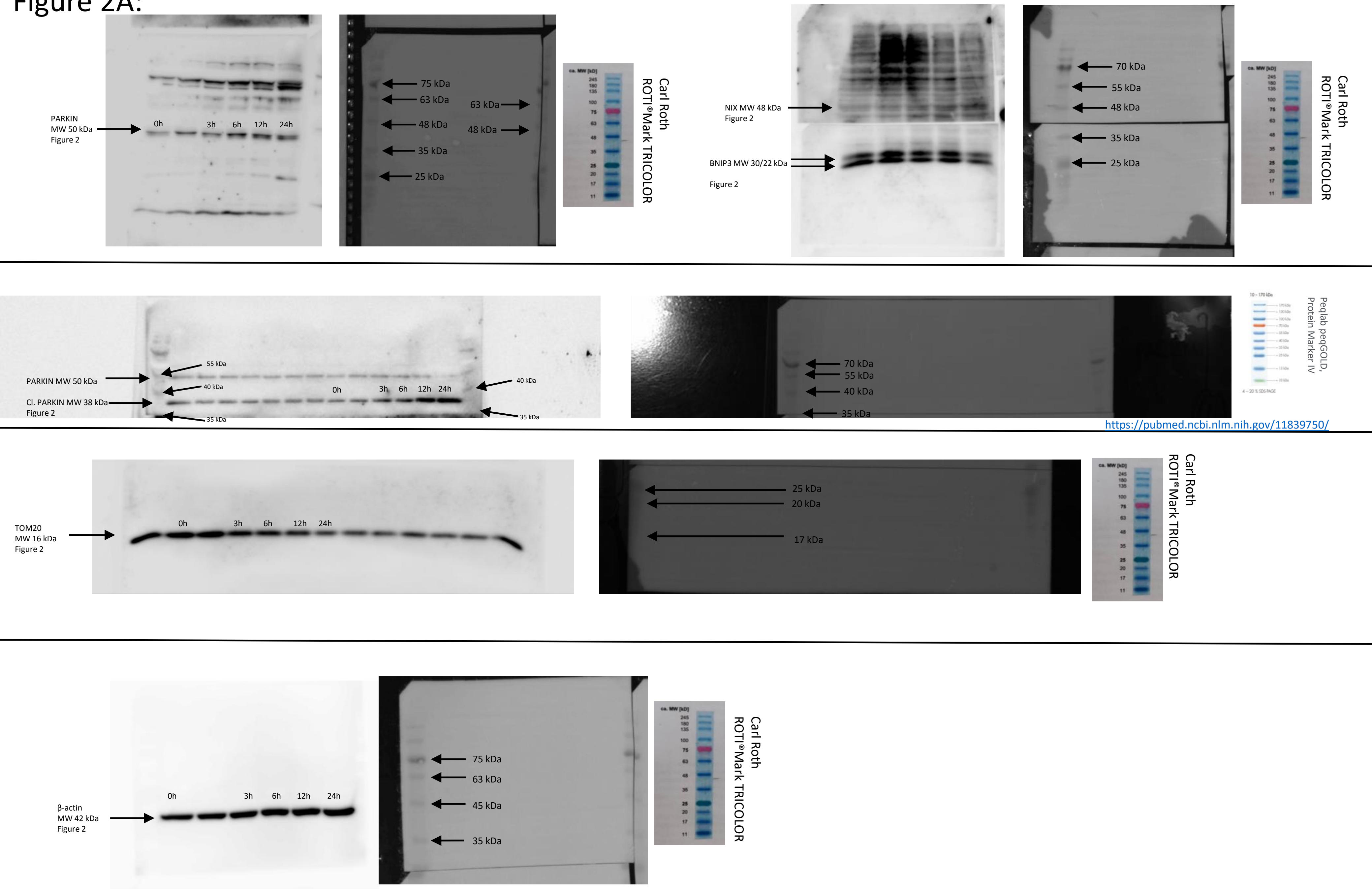
PARKIN
MW 50 kDa
Figure 2
0h
3h
6h
12h
24h
75 kDa
63 kDa
48 kDa
35 kDa
25 kDa
63 kDa
48 kDa
Carl Roth
ROTI®Mark TRICOLOR
NIX MW 48 kDa
Figure 2
BNIP3 MW 30/22 kDa
Figure 2
70 kDa
55 kDa
48 kDa
35 kDa
25 kDa
Carl Roth
ROTI®Mark TRICOLOR
PARKIN MW 50 kDa
Cl. PARKIN MW 38 kDa
Figure 2
55 kDa
40 kDa
35 kDa
0h
3h
6h
12h
24h
40 kDa
35 kDa
70 kDa
55 kDa
40 kDa
35 kDa
Peqlab peqGOLD,
Protein Marker IV
https://pubmed.ncbi.nlm.nih.gov/11839750/
TOM20
MW 16 kDa
Figure 2
0h
3h
6h
12h
24h
25 kDa
20 kDa
17 kDa
Carl Roth
ROTI®Mark TRICOLOR
β-actin
MW 42 kDa
Figure 2
0h
3h
6h
12h
24h
75 kDa
63 kDa
45 kDa
35 kDa
Carl Roth
ROTI®Mark TRICOLOR

Figure 3A:

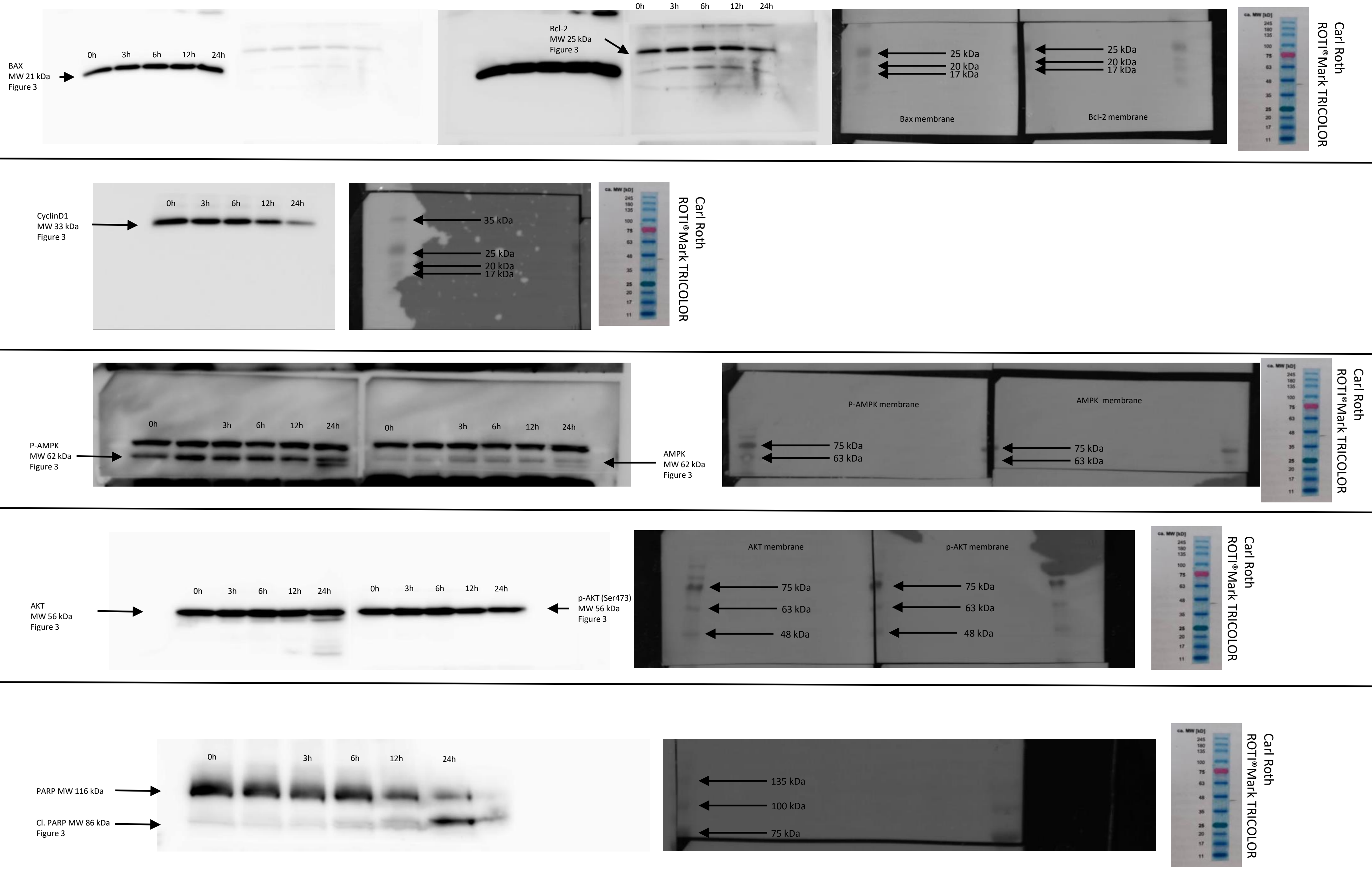
BAX
MW 21 kDa
Figure 3
0h 3h 6h 12h 24h
Bcl-2
MW 25 kDa
Figure 3
0h 3h 6h 12h 24h
25 kDa
20 kDa
17 kDa
Bax membrane
Bcl-2 membrane
Carl Roth
ROTI®Mark TRICOLOR
CyclinD1
MW 33 kDa
Figure 3
0h 3h 6h 12h 24h
35 kDa
25 kDa
20 kDa
17 kDa
Carl Roth
ROTI®Mark TRICOLOR
P-AMPK
MW 62 kDa
Figure 3
0h 3h 6h 12h 24h
0h 3h 6h 12h 24h
AMPK
MW 62 kDa
Figure 3
P-AMPK membrane
AMPK membrane
75 kDa
63 kDa
Carl Roth
ROTI®Mark TRICOLOR
AKT
MW 56 kDa
Figure 3
0h 3h 6h 12h 24h
0h 3h 6h 12h 24h
p-AKT (Ser473)
MW 56 kDa
Figure 3
AKT membrane
p-AKT membrane
75 kDa
63 kDa
48 kDa
Carl Roth
ROTI®Mark TRICOLOR
PARP MW 116 kDa
Cl. PARP MW 86 kDa
Figure 3
0h 3h 6h 12h 24h
135 kDa
100 kDa
75 kDa
Carl Roth
ROTI®Mark TRICOLOR

Figure 3A:

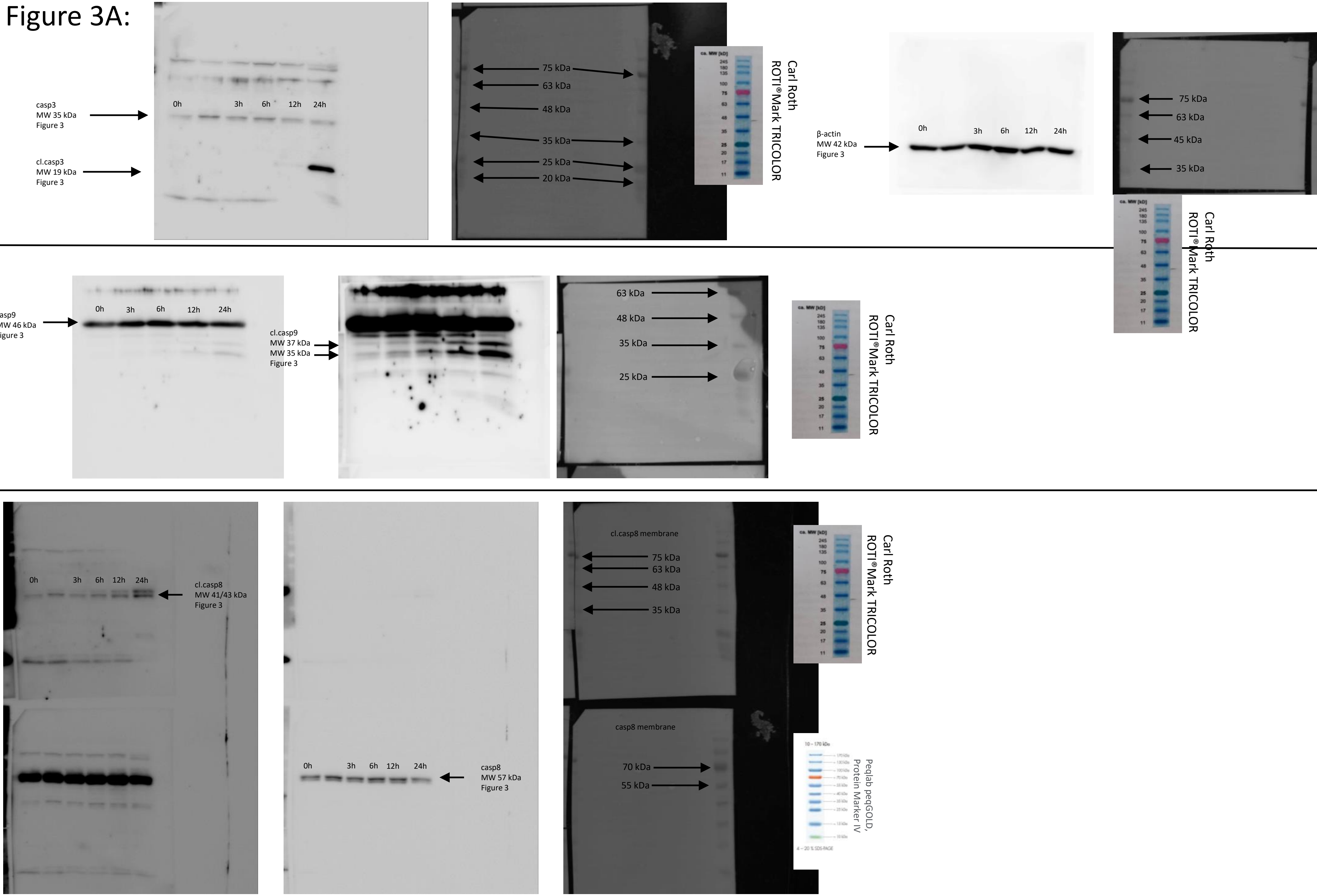

casp3
MW 35 kDa
Figure 3
cl.casp3
MW 19 kDa
Figure 3
0h
3h
6h
12h
24h
75 kDa
63 kDa
48 kDa
35 kDa
25 kDa
20 kDa
Carl Roth
ROTI®Mark TRICOLOR
β-actin
MW 42 kDa
Figure 3
0h
3h
6h
12h
24h
75 kDa
63 kDa
45 kDa
35 kDa
Carl Roth
ROTI®Mark TRICOLOR
casp9
MW 46 kDa
Figure 3
0h
3h
6h
12h
24h
cl.casp9
MW 37 kDa
MW 35 kDa
Figure 3
63 kDa
48 kDa
35 kDa
25 kDa
Carl Roth
ROTI®Mark TRICOLOR
0h
3h
6h
12h
24h
cl.casp8
MW 41/43 kDa
Figure 3
0h
3h
6h
12h
24h
casp8
MW 57 kDa
Figure 3
cl.casp8 membrane
75 kDa
63 kDa
48 kDa
35 kDa
casp8 membrane
70 kDa
55 kDa
Carl Roth
ROTI®Mark TRICOLOR
Peqlab peqGOLD,
Protein Marker IV

Figure 4A:

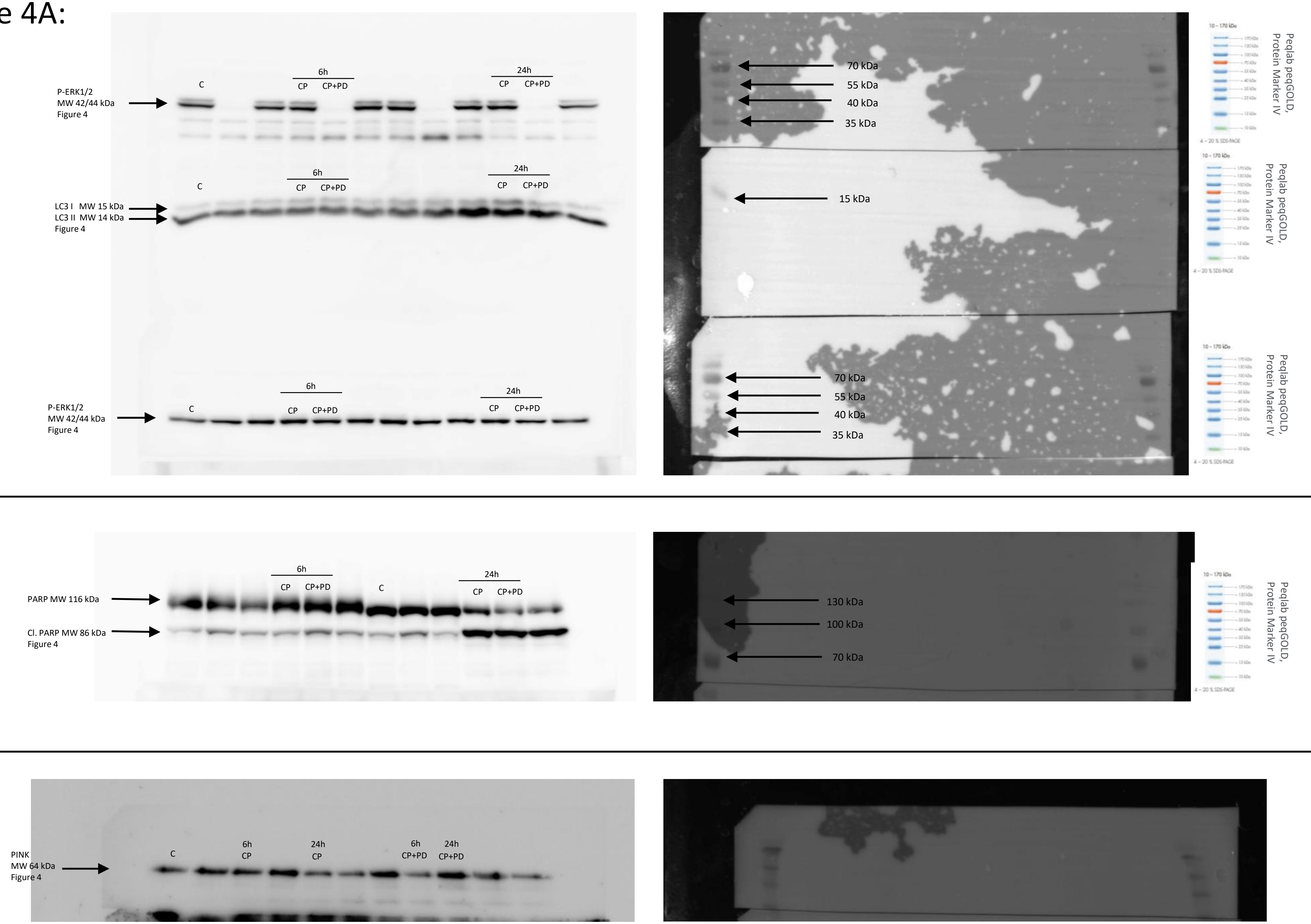

P-ERK1/2
MW 42/44 kDa
Figure 4
C
6h
CP
CP+PD
24h
CP
CP+PD
LC3 I MW 15 kDa
LC3 II MW 14 kDa
Figure 4
C
6h
CP
CP+PD
24h
CP
CP+PD
P-ERK1/2
MW 42/44 kDa
Figure 4
C
6h
CP
CP+PD
24h
CP
CP+PD
70 kDa
55 kDa
40 kDa
35 kDa
15 kDa
70 kDa
55 kDa
40 kDa
35 kDa
Peqlab peqGOLD, Protein Marker IV
Peqlab peqGOLD, Protein Marker IV
Peqlab peqGOLD, Protein Marker IV
PARP MW 116 kDa
Cl. PARP MW 86 kDa
Figure 4
6h
CP
CP+PD
C
24h
CP
CP+PD
130 kDa
100 kDa
70 kDa
Peqlab peqGOLD, Protein Marker IV
PINK
MW 64 kDa
Figure 4
C
6h
CP
24h
CP
6h
CP+PD
24h
CP+PD

Figure 4A:

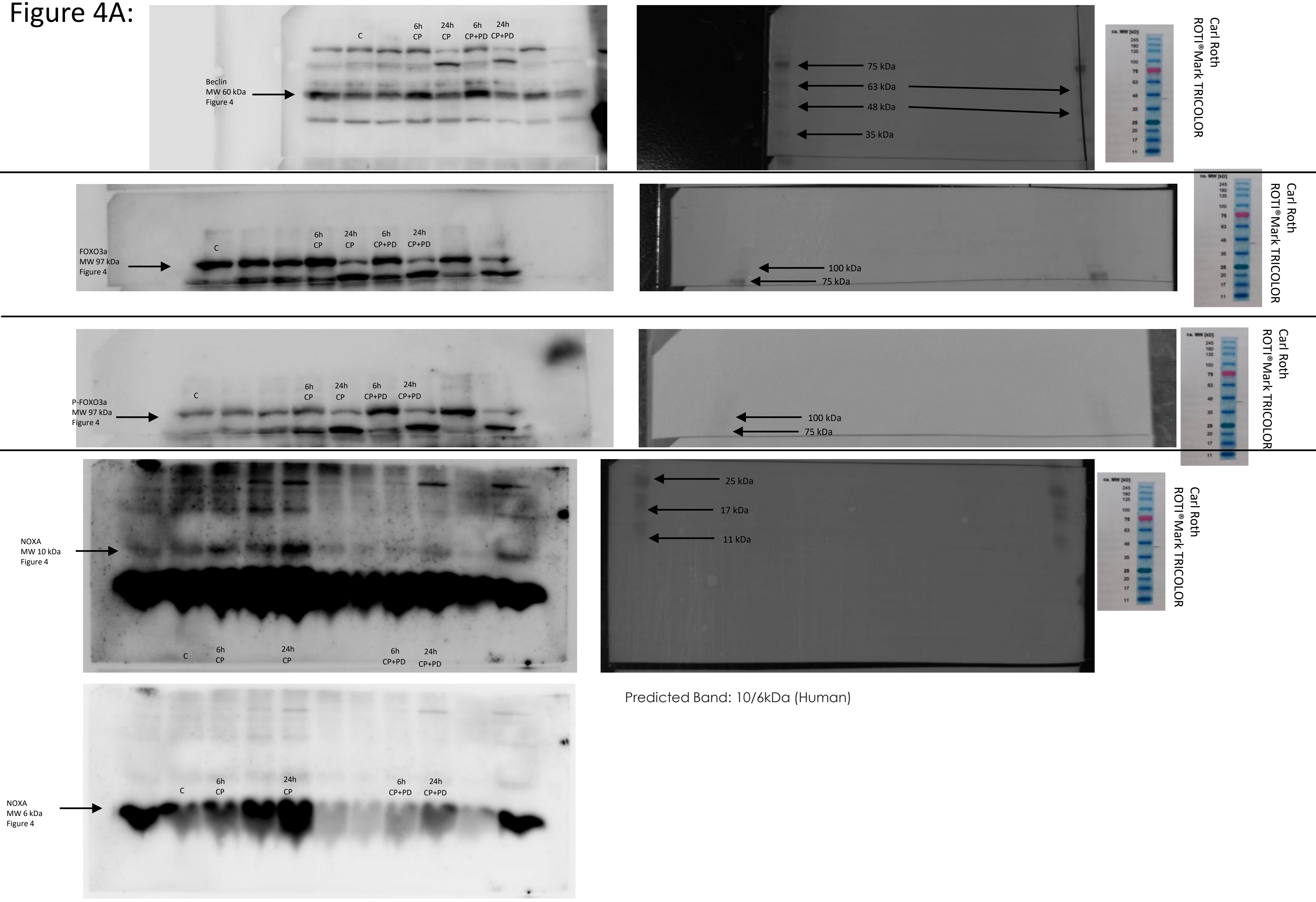
C
6h CP
24h CP
6h CP+PD
24h CP+PD
Beclin
MW 60 kDa
Figure 4
75 kDa
63 kDa
48 kDa
35 kDa
Carl Roth
ROTI®Mark TRICOLOR
FOXO3a
MW 97 kDa
Figure 4
100 kDa
75 kDa
P-FOXO3a
MW 97 kDa
Figure 4
NOXA
MW 10 kDa
Figure 4
25 kDa
17 kDa
11 kDa
NOXA
MW 6 kDa
Figure 4
Predicted Band: 10/6kDa (Human)

Figure 4A:

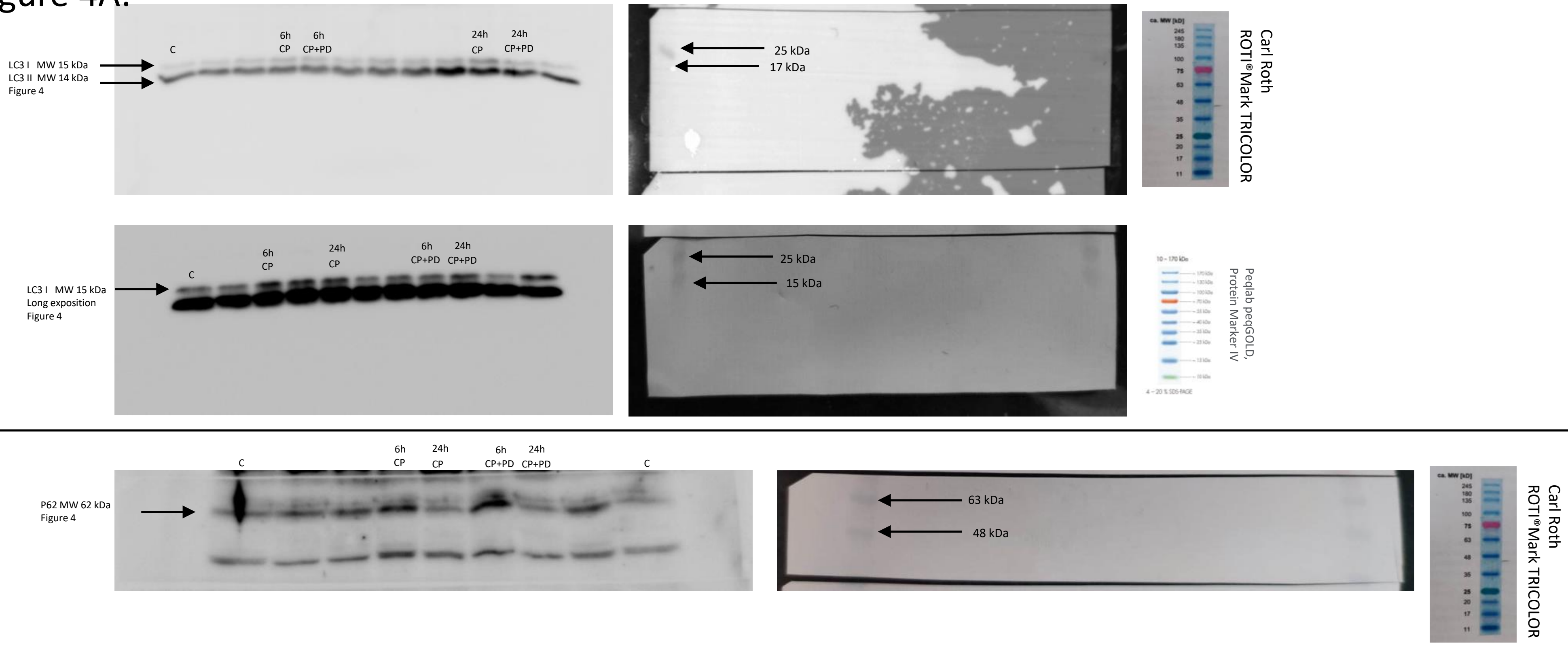

C
6h CP
6h CP+PD
24h CP
24h CP+PD
LC3 I MW 15 kDa
LC3 II MW 14 kDa
Figure 4
25 kDa
17 kDa
Carl Roth
ROTI®Mark TRICOLOR
C
6h CP
24h CP
6h CP+PD
24h CP+PD
LC3 I MW 15 kDa
Long exposition
Figure 4
25 kDa
15 kDa
Peqlab peqGOLD,
Protein Marker IV
C
6h CP
24h CP
6h CP+PD
24h CP+PD
C
P62 MW 62 kDa
Figure 4
63 kDa
48 kDa
Carl Roth
ROTI®Mark TRICOLOR

Figure 6F:

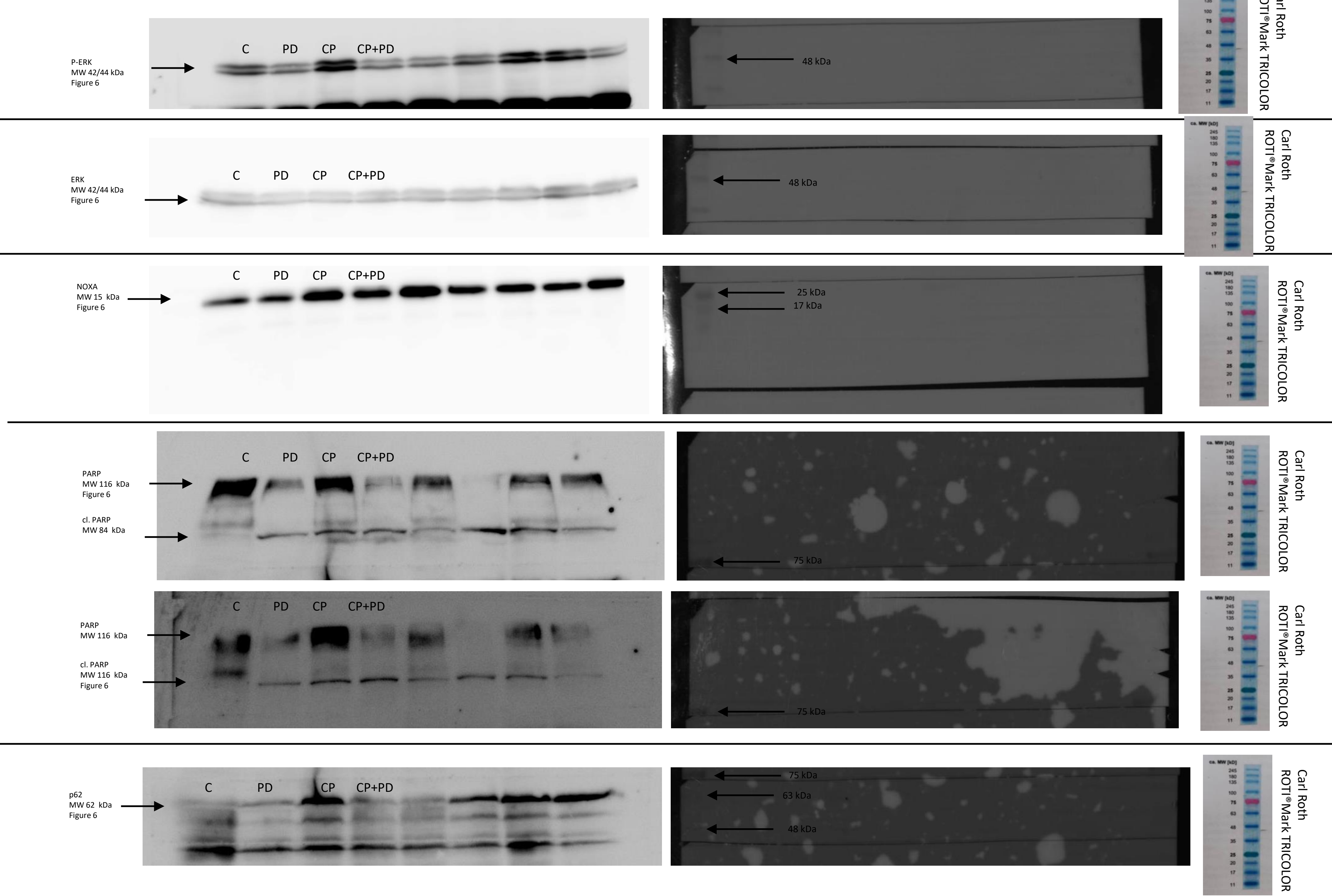

P-ERK
MW 42/44 kDa
Figure 6
C
PD
CP
CP+PD
48 kDa
Carl Roth
ROTI®Mark TRICOLOR
ERK
MW 42/44 kDa
Figure 6
C
PD
CP
CP+PD
48 kDa
Carl Roth
ROTI®Mark TRICOLOR
NOXA
MW 15 kDa
Figure 6
C
PD
CP
CP+PD
25 kDa
17 kDa
Carl Roth
ROTI®Mark TRICOLOR
PARP
MW 116 kDa
Figure 6
cl. PARP
MW 84 kDa
C
PD
CP
CP+PD
75 kDa
Carl Roth
ROTI®Mark TRICOLOR
PARP
MW 116 kDa
cl. PARP
MW 116 kDa
Figure 6
C
PD
CP
CP+PD
75 kDa
Carl Roth
ROTI®Mark TRICOLOR
p62
MW 62 kDa
Figure 6
C
PD
CP
CP+PD
75 kDa
63 kDa
48 kDa
Carl Roth
ROTI®Mark TRICOLOR

Beclin
MW 60 kDa
Figure 6

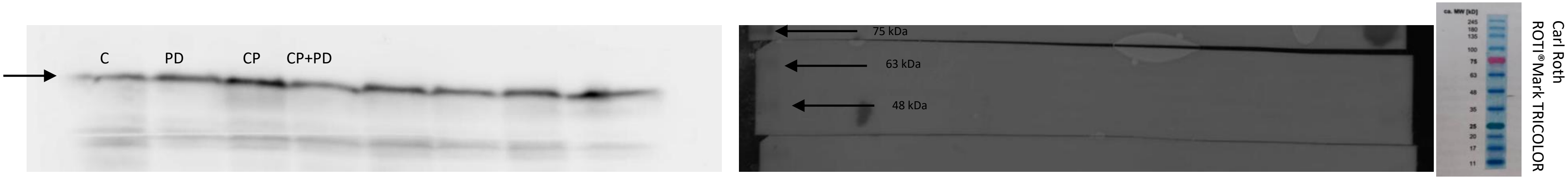

LC3 I/II
MW 15 kDa
MW 14 kDa
Figure 6

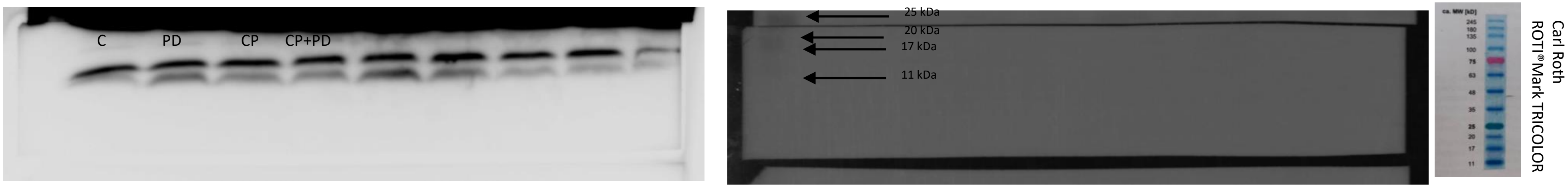

TOM20
MW 16 kDa
Figure 6

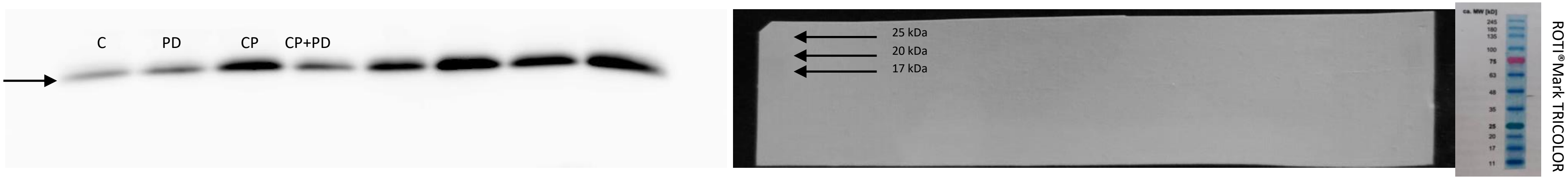

PINK
MW50 kDa
Figure 6

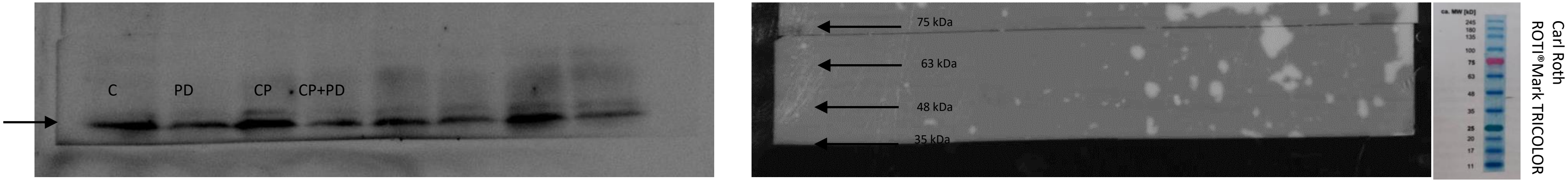

GAPDH
MW37 kDa
Figure 6

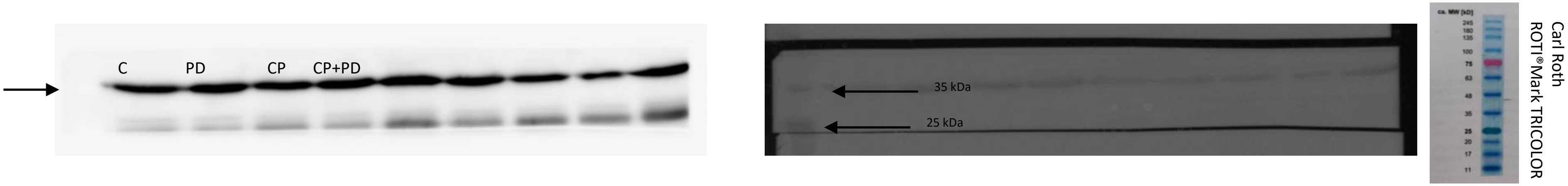

Figure 1H:

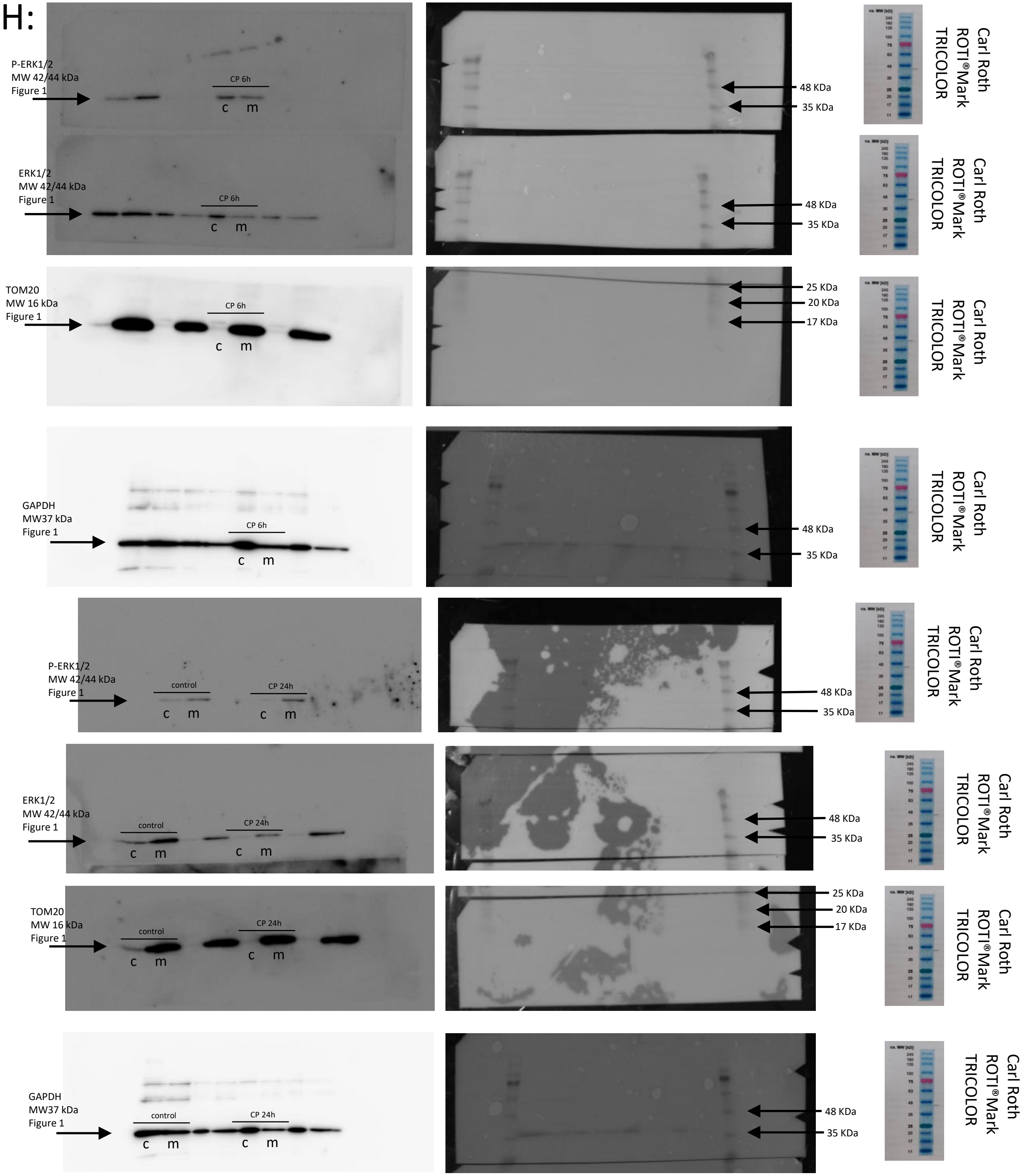

P-ERK1/2
MW 42/44 kDa
Figure 1
CP 6h
c m
48 KDa
35 KDa
Carl Roth ROTI®Mark TRICOLOR
ERK1/2
MW 42/44 kDa
Figure 1
CP 6h
c m
48 KDa
35 KDa
TOM20
MW 16 kDa
Figure 1
CP 6h
c m
25 KDa
20 KDa
17 KDa
GAPDH
MW37 kDa
Figure 1
CP 6h
c m
48 KDa
35 KDa
P-ERK1/2
MW 42/44 kDa
Figure 1
control
CP 24h
c m
c m
48 KDa
35 KDa
ERK1/2
MW 42/44 kDa
Figure 1
control
CP 24h
c m
c m
48 KDa
35 KDa
TOM20
MW 16 kDa
Figure 1
control
CP 24h
c m
c m
25 KDa
20 KDa
17 KDa
GAPDH
MW37 kDa
Figure 1
control
CP 24h
c m
c m
48 KDa
35 KDa
